# Oxygen vacancies in Ru/TiO_2_ - drivers of low-temperature CO_2_ methanation assessed by multimodal operando spectroscopy

**DOI:** 10.1016/j.isci.2022.103886

**Published:** 2022-02-08

**Authors:** Sebastian Cisneros, Ali Abdel-Mageed, Jawaher Mosrati, Stephan Bartling, Nils Rockstroh, Hanan Atia, Hayder Abed, Jabor Rabeah, Angelika Brückner

**Affiliations:** 1 Leibniz-Institut für Katalyse, Albert-Einstein-Str. 29A, 18059 Rostock, Germany; 2 Department of Chemistry, Faculty of Science, Cairo University, Giza 12613, Egypt; 3 Laboratoire de chimie des matériaux et catalyse, Département de chimie, Faculté des sciences de Tunis, Université de Tunis el Manar, Tunis 1092, Tunisie; 4 Department Life, Light and Matter, University of Rostock, Albert-Einstein-Str. 25, 18059 Rostock, Germany

**Keywords:** Chemistry, Inorganic chemistry, Catalysis

## Abstract

Hydrogenation of CO_2_ is very attractive for transforming this greenhouse gas into valuable high energy density compounds. In this work, we developed a highly active and stable Ru/TiO_2_ catalyst for CO_2_ methanation prepared by a solgel method that revealed much higher activity in methanation of CO_2_ (ca. 4–14 times higher turnover frequencies at 140–210°C) than state-of-the-art Ru/TiO_2_ catalysts and a control sample prepared by wetness impregnation. This is attributed to a high concentration of O-vacancies, inherent to the solgel methodology, which play a dual role for 1) activation of CO_2_ and 2) transfer of electrons to interfacial Ru sites as evident from operando DRIFTS and *in situ* EPR investigations. These results suggest that charge transfer from O-vacancies to interfacial Ru sites and subsequent electron donation from filled metal d-orbitals to antibonding orbitals of adsorbed CO are decisive factors in boosting the CO_2_ methanation activity.

## Highlights

• Solgel prepared Ru/TiO_2_ outperforms methanation activity of similar materials
• Reliable insight of O-vacancies role is gained by combined operando techniques
• Enhanced interaction of O-vacancy-Ru^0^ sites boosts methane rate

## Graphical abstract

**Figure:**
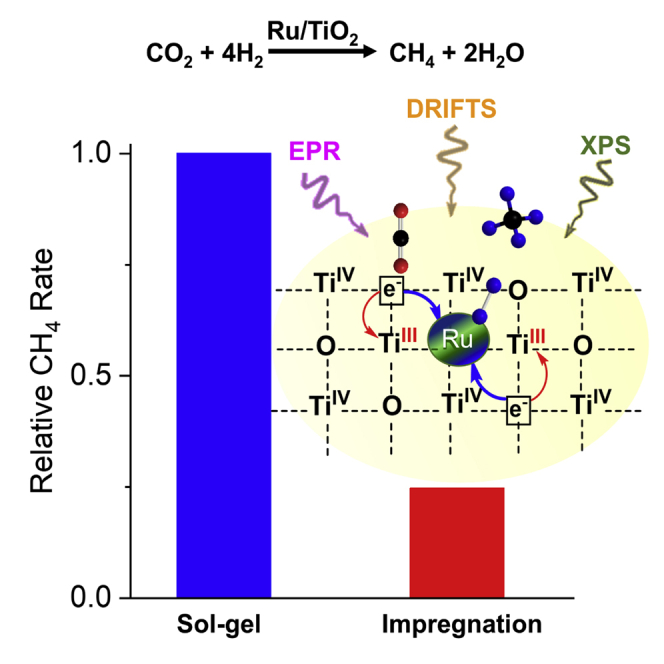

## Introduction

Valorizing a problematic greenhouse gas such as CO_2_ by chemical storage of renewable hydrogen in the form of high energy density compounds is an order of the day to face climate change (Lee et al., 2020; Li et al., 2018; Porosoff et al., 2016; Wang et al., 2011). In particular, the selective methanation of CO_2_ is anticipated to improve the storage and distribution of renewable hydrogen using the natural gas grid, i.e., the power to gas (P2G) process. It is hence of great interest from a fundamental as well as from a technological perspective to develop better catalysts for this reaction (De et al., 2020). Because the most active catalysts in this process include precious group VIII elements (Mills and Steffgen, 1974), it is essential to gain a deep understanding about the interactions between dispersed active entities and the supporting matrix (Campbell, 2012; Tauster et al., 1981) to overcome the limitations, e.g., high costs, placed to such noble metals when pretended to be used in technical high scale applications (Moioli and Züttel, 2020).

Important advances in the elucidation of geometric and/or electronic structural properties that influence the reduction of CO_2_ on Ru based catalysts have been reported. In particular, the paramount role of the support reducibility and the related formation of oxygen vacancies have been recognized. It has been proposed that O-vacancies promote electronic metal-support interactions (EMSIs) in Ru/TiO_2_ catalysts by partial encapsulation of Ru NPs within a layer of reduced TiO_2_, supposed to enhance the rate of methane formation (Abdel-Mageed et al., 2020). The reason for this effect has been attributed to changes in the local charge density of the Ru sites as well as to an increase in CO_2_ adsorption sites, as more O-vacancies can be produced. In addition, in other catalysts such as Ru/ZrO_2_, O-vacancies were found to be responsible for electron transfer from the support to the Ru NPs. This strengthened the Ru-CO bonding which in turn enhanced the methane formation rate, yet without encapsulation of the Ru NPs by reduced support layers (Chen et al., 2021). Besides their role in the stabilization of the Ru-CO bonding, O-vacancies are considered also as adsorption sites for CO_2_ leading to the formation of carbonates, bicarbonates (Guo et al., 2018; He et al., 1985), and/or formates. The latter are regarded as key intermediates in the pathway to CH_4_ (Falbo et al., 2019; Wang et al., 2015, 2016a, 2017). All these observations demonstrate the key role of such defects in reducible supports for methane production, which is assumed to proceed via the reaction sequence: CO_2_ → Carbonate/Formate → (CO_ad_) → CH_4_, although the precise mechanism is still under debate (Kattel et al., 2017; Sápi et al., 2018; Yan et al., 2019).

The aim of this study is to tailor the EMSI in Ru/TiO_2_ catalysts toward improved catalytic performance in methanation of CO_2_ with low Ru content. Because we previously found that sol-gel synthesis is very beneficial to prepare supported redox-active metal catalysts with improved catalytic performance that was related to the key role of O-vacancies in the catalytic cycle (Mosrati et al., 2021), this synthesis method has been employed to produce a Ru/TiO_2_ catalyst with a targeted Ru loading below 1 wt%. Moreover, this synthesis route was selected because it allows us to control catalyst structure and composition (Ward and Ko, 1995) in a way that facilitates correlation between activity and morphology and revealed to be very versatile in the bottom-up synthesis of heterogeneous catalysts with tailored properties for a variety of reactions (Debecker, 2018).

Our new 0.90 wt % Ru/TiO_2_-SG catalyst enabled methanation rates that are, to the best of our knowledge, unprecedented values for CO_2_ methanation below 200°C on Ru/TiO_2_ catalysts. The turnover frequency (TOF) was at least 4-times higher than that reported for other Ru/TiO_2_ catalysts under similar conditions and with higher Ru contents. This much superior methane activity is probably driven by the creation of special vacancy-Ru^0^ sites at the metal-support perimeter interface which are obviously created by the solgel synthesis method, in contrast to catalysts prepared by other conventional methods, that were much less active, despite a significantly higher Ru content. This demonstrates clearly that it is not mainly the net noble metal content which governs catalytic performance, but unique structural and electronic effects that create superior activity of such tiny metal species.

To unravel structure-reactivity relationships, we used an array of operando, *in situ* and *ex situ* characterization techniques such as N_2_ physisorption, X-ray diffraction (XRD), Raman spectroscopy, high angle annular dark field scanning transmission electron microscopy (HAADF-STEM), diffuse reflectance Fourier transform spectroscopy (DRIFTS), near-ambient X-ray photoelectron spectroscopy (NAP-XPS), and electron paramagnetic resonance (EPR). Thus, we could show that enhanced electronic Ru-support interactions are able to boost low-temperature methanation activity beyond that of state-of-the-art methanation catalysts.

## Results and discussion

### Catalysts characterization

The Ru content in the Ru/TiO_2_-SG catalyst (0.90 wt %, prepared by solgel methodology) and the Ru/TiO_2_-Imp control sample (0.91 wt %, prepared by the incipient wetness impregnation and annealing in air at 500°C) was virtually the same, as determined from ICP assessments.

**Figure 1:**
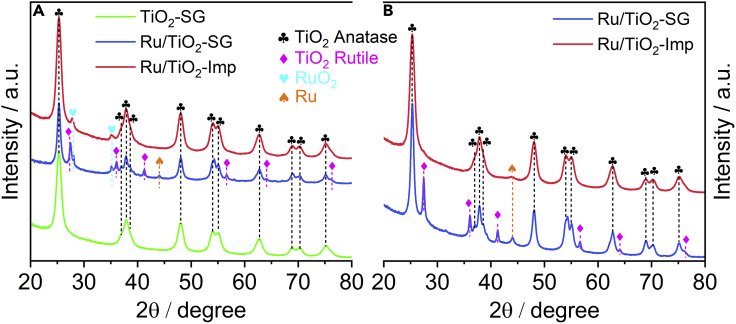
Catalyst characterization: XRD patterns (A) XRD powder patterns of the fresh TiO_2_ support and fresh Ru catalysts. (B) XRD powder patterns of Ru catalysts after 6h reaction in H_2_:CO_2_ = 4:1 flow up to 270°C.

The XRD powder pattern of the bare support prepared by the solgel method (TiO_2_-SG) shows only typical reflections of the anatase (101), (004), and (200) planes at 2θ = 25.3°, 37.9, and 48.0°, respectively (ICDD 01-075-2547, Figure 1A). The pattern of the Ru/TiO_2_-SG catalyst shows additional reflections of rutile TiO_2_ at 2θ = 27.4°, 36.1, and 41.2° corresponding to planes (110), (101), and (111) (ICDD 00-021-1276), respectively. This phase change has been ascribed to the similarity between the lattice constants of RuO_2_ and rutile TiO_2_ (Wang and Gordon, 2013). This was also reported by Kim et al. who detected the formation of rutile TiO_2_ upon coverage of mesoporous TiO_2_ (anatase) with RuO_2_ nanoparticles after annealing at 250°C (Kim et al., 2019). In contrast, no rutile formation was observed in the Ru/TiO_2_-Imp catalyst. This suggests that intimate mixing of TiO_2_ and Ru precursors, which is typical for the solgel method, promotes incorporation of Ru in Ti lattice positions of TiO_2_ and induces crystal phase transitions. The weight fraction of rutile TiO_2_ in the fresh Ru/TiO_2_-SG (calculated by the method of Zhang and Banfield (Zhang and Banfield, 2000)) amounts to 18.6 wt %, which is similar to commercial P-25 (80 wt % anatase, 20 wt % rutile) (Sassoye et al., 2011). Reflections of RuO_2_ (110) and (101) planes (ICDD 01-088-0322) at 2θ = 28.1 and 35.1° are present in both as-prepared Ru catalysts. The spent catalysts after reaction (up to 270°C, H_2_:CO_2_ = 4:1) do not show any RuO_2_ reflections (Figure 1B); however, the reflections of TiO_2_ rutile (19 wt %) are still seen. Metallic Ru^0^ crystallites are evidenced by a small peak at 2θ = 44.1° which is most pronounced for the spent Ru/TiO_2_-SG sample.

*Ex situ* Raman spectra of the fresh TiO_2_ and catalyst samples are shown in Figure S1. Typical vibration modes of anatase at 144, 196, 397, 514, and 639 cm^−1^ are seen in the spectrum of the bare support (Zhang et al., 2000). For the Ru/TiO_2_-SG catalyst, a broadening of the main signal and a slight shift to about 141 cm^−1^ was observed, besides weak signals at 447 and 611 cm^−1^, associated with the E_g_ and A_1g_ vibration modes of planar O-O and Ti-O stretching in rutile, respectively (Ma et al., 2007; Zhang et al., 1995). Such weak R-E_g_ and R-A_1g_ signals are not evident in the spectrum of the Ru/TiO_2_-Imp catalyst, which, in agreement with XRD (Figure 1A) resembles that of bare anatase TiO_2_.

N_2_ isotherms of all samples show the typical type IV shape with a hysteresis loop characteristic of mesopores. Pore volume and pore area as a function of the pore size are plotted in Figure S2. The mean pore diameters, pore volumes, and BET surface areas (BET SSA) are stated in Table 1.

**Table 1:** Pore and surface properties of the fresh support and catalysts

Sample	Property		
	Mean pore diameter (nm)	Pore volume (cm^3^ g^−1^)	BET SSA (m^2^ g^−1^)
Bare TiO_2_-SG	5	0.26	141
Ru/TiO_2_-SG (fresh)	8–9	0.17	61
Ru/TiO_2_-Imp (fresh)	12	0.43	136

The bare TiO_2_-SG support shows a narrow pore size distribution centered at ca. 5 nm (Figure S2D), a pore volume of 0.26 cm^3^ g^-1^, and a BET SSA of 141 m^2^ g^−1^. The latter two values are much lower for the fresh Ru/TiO_2_-SG catalyst, the pore size distribution is slightly broader and centered at 8–9 nm (Figure S2E). This suggests that incorporation of Ru may have caused a partial disruption of the mesoscopic structure. For the impregnated control sample Ru/TiO_2_-Imp, BET SSA dropped slightly to 136 m^2^ g^−1^ while the pore diameter and volume increased significantly (Figure S2F) in comparison to the bare support and the Ru/TiO_2_-SG catalyst (Table 1). Considering the fact that all samples including the bare support have experienced the same 3h pretreatment at 500°C in air, it can be assumed that the marked differences in BET SSA and pore volume between Ru/TiO_2_-SG and Ru/TiO_2_-Imp (Table 1) are not caused by this thermal pretreatment but might be because of special interactions of the Ru component with the support resulting from the preparation route.

The reducibility of TiO_2_-SG and Ru/TiO_2_-SG samples has been evaluated by H_2_-TPR measurements (Figure S3). While the bare support showed only a weak single peak at ca. 552°C, the Ru/TiO_2_-SG catalyst shows several H_2_ consumption peaks between 170 and 520°C, resulting from Ru species which differ in their oxidation states and/or the strength of their interaction with the carrier. The total consumed amount of H_2_ was ca. 9 times higher than the stoichiometric amount that would be needed to reduce all Ru^4+^ in the sample to Ru^0^ (given that all reducible Ru was present as RuO_2_). This means that not only Ru^n+^ species but also Ti^4+^ ions from the support were reduced. This may suggest that, after reducing RuO_x_ species, H_2_ is split into atoms on the surface of the formed Ru^0^ particles from which they spill over to the metal-support interface where they react with lattice oxygen to form water. This should lead to partial reduction of the support and would agree with previous observations on supported metal/TiO_2_ catalysts in which the reduction of Ti^4+^ was promoted by the vicinity of the metal. A similar effect has been observed for Au/TiO_2_ catalysts (Zhang et al., 2021). In contrast to the solgel derived catalyst, sample Ru/TiO_2_-Imp shows only a narrow reduction peak at 157°C and the amount of consumed H_2_ is much lower, ca. 21% of that observed for Ru/TiO_2_-SG. The reason may be that in this case the interaction between Ru/RuO_x_ and TiO_2_ is much weaker than in Ru/TiO_2_-SG.

### Catalytic tests and kinetic analysis

**Figure 2:**
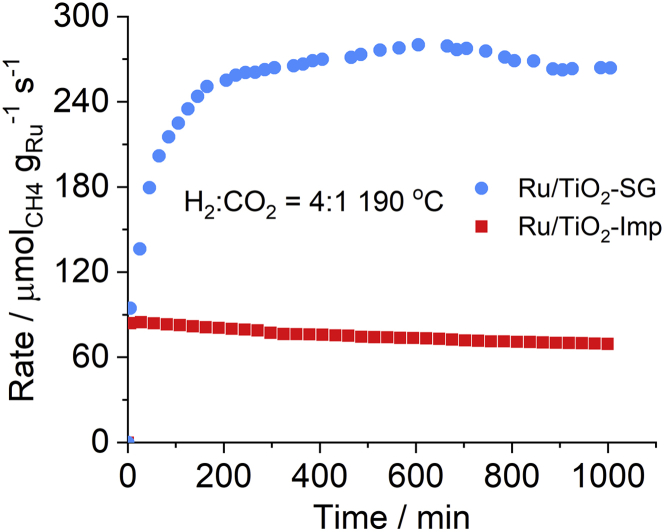
Catalytic test and kinetic analysis at 190°C Ru mass normalized methane rate on Ru/TiO_2_-SG and Ru/TiO_2_-Imp assessed at 190°C. Gas mixture: H_2_:CO_2_ = 4:1 (24 mL min^−1^ H_2_, 6 mL min^−1^ CO_2_).

**Figure 3:**
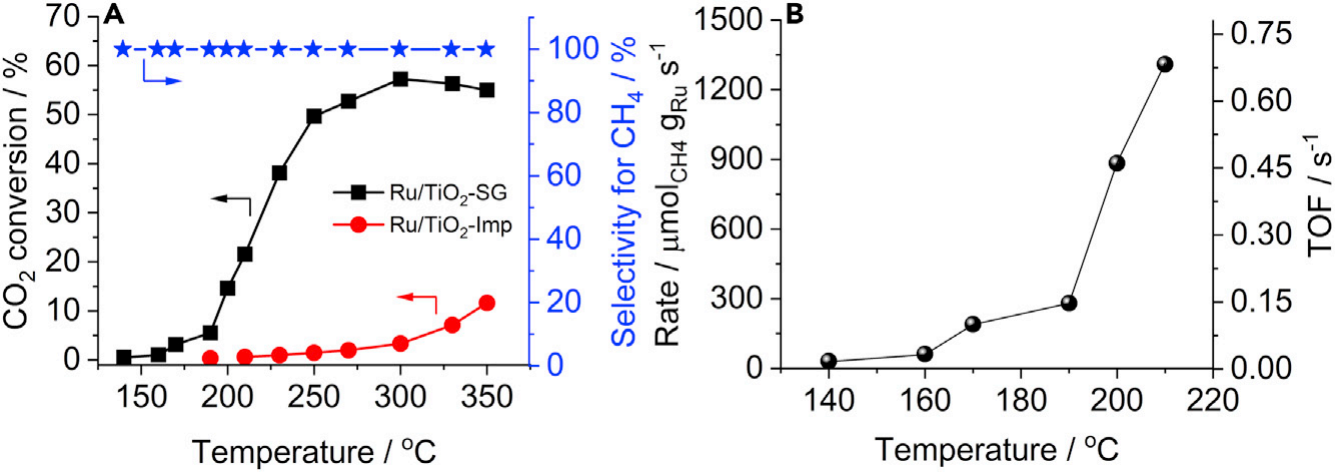
Catalytic test and kinetic analysis at different temperatures (A) CO_2_ conversion and CH_4_ selectivity on Ru/TiO_2_-SG and Ru/TiO_2_-Imp. (B) CH_4_ formation rates normalized on the Ru mass and TOF values on Ru/TiO_2_-SG. Gas mixture: H_2_:CO_2_ = 4:1 (32 mL min^−1^ H_2_, 8 mL min^−1^ CO_2_).

We first examined the rates of CH_4_ formation normalized on the Ru mass (Equation 1) at 190°C on both catalysts during ca. 1000 min (17 h) on stream (Figure 2). Catalyst Ru/TiO_2_-SG passed an activation period of about 200 min after which it reached a roughly three times higher CH_4_ formation rate than the impregnated control catalyst Ru/TiO_2_-Imp. Remarkably, the latter did not show such activation though such an effect has been observed previously also for other Ru/TiO_2_ catalysts (Abdel-Mageed et al., 2020) in which it was ascribed to the reduction of oxidized Ru and the creation of O-vacancies in the support. Furthermore, we also performed temperature screening tests from 140–350°C (Figure 3A). The selectivity to CH_4_ in this temperature window was always 100% for both catalysts, according to Equation 3. The much higher performance of the Ru/TiO_2_-SG catalyst, despite the same Ru loading, might be because of a higher dispersion and surface exposition of Ru NPs and a more effective electron transfer from O-vacancies to interfacial Ru sites. This issue will be discussed below in more detail.

We next focus our interest on benchmarking the activity of our solgel catalyst against catalysts from previous relevant reports (Abdel-Mageed et al., 2020, 2021; Abe et al., 2009; Chai et al., 2019; Kim et al., 2016, 2018; Lin et al., 2014, 2017; Petala and Panagiotopoulou, 2018; Sassoye et al., 2011) (Table S1). The methane formation rates calculated according to Equation 1 at differential conditions between 140°C and 210°C are shown in Figure 3B. Already at 200°C the Ru mass normalized CH_4_ formation rate of our catalyst was ca. 884 μmol_CH4_ g_Ru_^−1^ s^−1^, i.e., around 7 times higher than values reported for a Ru/TiO_2_(P-25) (Sassoye et al., 2011). Furthermore, when compared to a more recent report regarding CO_2_ methanation on Ru/TiO_2_(P-90) and Ru/TiO_2_(P-25) at 190°C (Abdel-Mageed et al., 2021), the activity of our Ru/TiO_2_-SG (ca. 264 μmol_CH4_ g_Ru_^−1^ s^−1^) is, respectively, between 2 and 5 times higher. At a first glance, these differences are surprising because the Ru content of our catalyst (ca. 0.9 wt %) is around 2 times lower than that used in those investigations (Abdel-Mageed et al., 2021; Sassoye et al., 2011). This calls for a more detailed consideration of turnover frequencies (TOF) provided below.

Even though TOFs are recommended for comparing more precisely the activity of catalysts, such an approach requires assumptions that can vary in different studies. For TOF calculations, the number of atoms exposed on the surface of the active metal NPs must be estimated. This estimation is not straightforward for polycrystalline materials and different values can be obtained depending on the selected plane(s) for the calculation (Shen et al., 2008). The procedure of Abe et al. (Abe et al., 2009) (Equation 5) used the number of atoms exposed on the Ru(001) plane in an hexagonal structure (ca. 1.739 ∗ 10^19^ atoms m^−2^) for TOF calculation of a Ru/TiO_2_ catalyst in CO_2_ methanation. For this calculation, the volume-area averaged mean diameter of the Ru NPs is needed. Using Equation 4, we determined a volume-area averaged diameter of 6.6 nm by evaluating ca. 300 Ru NPs from HAADF-STEM images similar to those presented in Figures S11A and S11B. The corresponding particle size distribution is shown in Figure S13. Using Equation 5 with N_Ru-atoms_ being the estimated total number of Ru atoms loaded on the surface of 1 g of catalyst, we obtained a value of 0.029 s^−1^ for CO_2_ methanation at 160 °C which corresponds to a TOF that is 3.4 times than that determined by Abe et al. for a 0.8 wt % Ru/TiO_2_ catalyst at the same reaction conditions. Further details about the calculation procedure can be found in the respective reference (Abe et al., 2009).

Alternatively, we used Equation 7 (Karim et al., 2009), for which no preferentially exposed crystal plane of the active metal must be specified. Instead, an averaged dispersion can be determined according to Equation 6. A TOF of 0.032 s^−1^ has been obtained for a dispersion of 19.3%. This TOF resembles that calculated by Equation 5. Therefore, we preferred Equation 7 to determine TOF values up to 210°C (Figure 3B), because this calculation is more suitable for polycrystalline materials. To the best of our knowledge, the obtained TOF values up to 210°C are the highest reported up to now for CO_2_ methanation in this temperature range. It appears that the adopted solgel method allows maximizing the methanation performance while decreasing the Ru loading. In the sections below we focus on determining the structural factors behind this promising catalytic behavior of Ru/TiO_2_-SG.

### *In situ* EPR measurements

**Figure 4:**
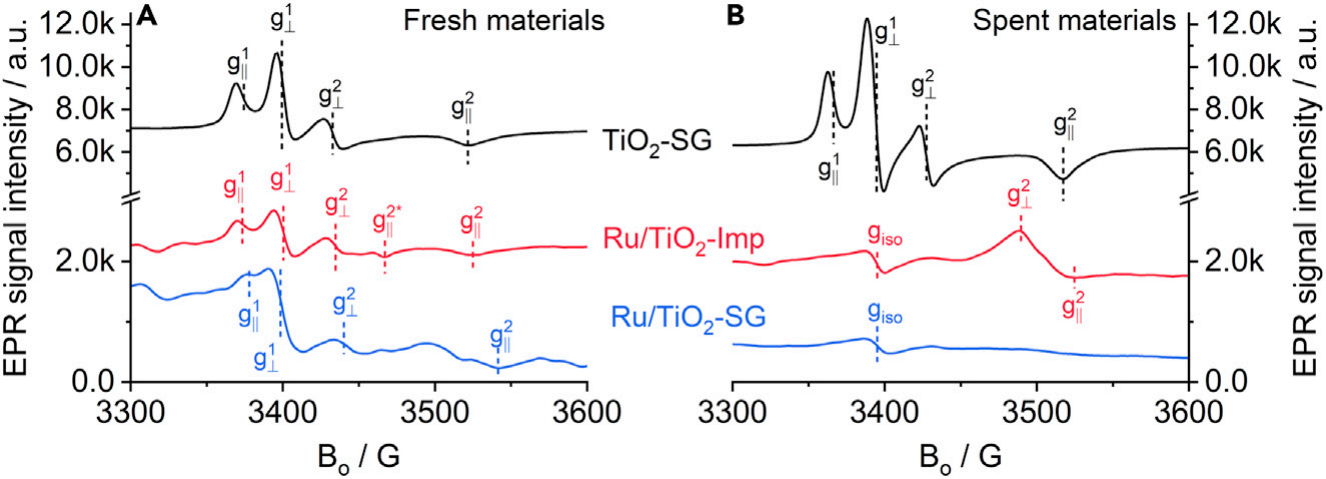
*In situ* EPR spectra recorded at −173°C for fresh and spent samples (A) Fresh samples as received in Ar. (B) Spent samples after 6h reaction up to 270°C in a flow of H_2_:CO_2_ = 4:1.

EPR spectroscopy is a unique technique to detect paramagnetic species that may form under reaction conditions, such as Ti^3+^ and inorganic radicals. Accordingly, EPR spectra of both catalysts as well as of the bare support were compared in fresh form under Ar flow before reaction and after 6h reaction in H_2_/CO_2_ flow up to 270°C (Figure 4). The EPR spectrum of the bare fresh TiO_2_ support shows the axial signal of a superoxide anion radical (O_2_^−^) with g^1^_||_ = 2.012 while the respective g^1^_⊥_ component may be superimposed on the typical signal from F-centers (an oxygen vacancy occupied by a single electron) around 2.000 (Coronado et al., 2001). In the spent Ru catalysts, this signal is denoted as g_iso_ and explicitly seen because it is not superimposed by O_2_^−^ (Figure 4B). O_2_^−^ species might have been formed by electron transfer from an F-center to O_2_ present as impurity in the Ar stream. In addition, the axial signal of Ti^3+^ with $g_{⊥}^{2}=1.984$ = 1.984 and $g_{∥}^{2}=1.930$ can be seen in bare fresh TiO_2_ which has been assigned to Ti^3+^ exposed on the surface (Chiesa et al., 2013; Howe and Gratzel, 1985; Livraghi et al., 2011; Mohajernia et al., 2020). Both signals are also seen in the spent bare support, yet in this case they are slightly more intense because of the reducing nature of the reaction atmosphere.

The EPR spectra of both fresh Ru/TiO_2_ catalysts show similar signals as the bare support but with lower intensity. This is probably because of partial electron transfer from F-centers and surface Ti^3+^ ions to Ru^4+^ species, which are in turn reduced to EPR-silent Ru^0^. This effect is even more evident for the spent catalysts and will be discussed below. Apart from the lower intensity, the general spectral shape of the Ru/TiO_2_-Imp catalyst is almost the same as for the bare support. An additional weak feature can be seen at $g_{∥}^{2*}=1.961$ while its perpendicular component might be superimposed at $g_{⊥}^{2}=1.982$. Such g values are characteristic for Ti^3+^ ions located at regular lattice positions of anatase with moderate tetragonal distortion (Mohajernia et al., 2020). i.e., they are embedded in the support matrix. In catalyst Ru/TiO_2_-SG catalyst, the signal of F-centers at g = 2.000 is more intense and the O_2_^−^ feature at $g_{∥}^{1}=2.010$ is weaker. Moreover, several poorly resolved features occur between $g_{⊥}^{2}=1.980$ and $g_{∥}^{2}=1.924$. As mentioned above, they may arise from $g_{∥}^{2}$ signals of Ti^3+^ ions with lower axial distortion embedded in lattice positions of TiO_2_. No signals from paramagnetic Ru^3+^ and/or Ru^+^ species (Valigi et al., 1985) could be observed in the fresh catalysts, indicating that Ru in these materials might be present as EPR silent Ru^4+^ and/or Ru^0^.

The EPR spectra of both spent Ru/TiO_2_ catalysts after ca. 6h of reaction at temperatures from 150 up to 270°C in H_2_/CO_2_ flow show only a weak line at g_iso_ = 2.000 from F-centers, whereas the axial signal of O_2_^−^ is not observed anymore (Figure 2B). In addition, the spent catalyst Ru/TiO_2_-Imp shows a pronounced signal of reduced Ti^3+^ species with $g_{⊥}^{2}=1.950$ and $g_{∥}^{2}=1.928$. Such a feature is entirely absent for catalyst Ru/TiO_2_-SG, as was also confirmed by *in situ* EPR spectra recorded during reaction (Figure S4). This suggests that in the most active catalyst Ru/TiO_2_-SG, electrons released in the anion vacancies after O removal are transferred preferentially to Ru^n+^ species at the metal-support interface, reducing them to EPR-silent Ru species. This is consistent with operando DRIFT and FTIR spectra of adsorbed CO (shown below) in which bands of carbonyl species associated with reduced Ru species were detected. In contrast, as evidenced by the pronounced Ti^3+^ EPR signal, these electrons are trapped partly by Ti^4+^ in the less active catalyst Ru/TiO_2_-Imp, which might limit their transfer to Ru^n+^, leading to the formation of active Ru^0^ species.

### Operando and *in situ* DRIFTS measurements

**Figure 5:**
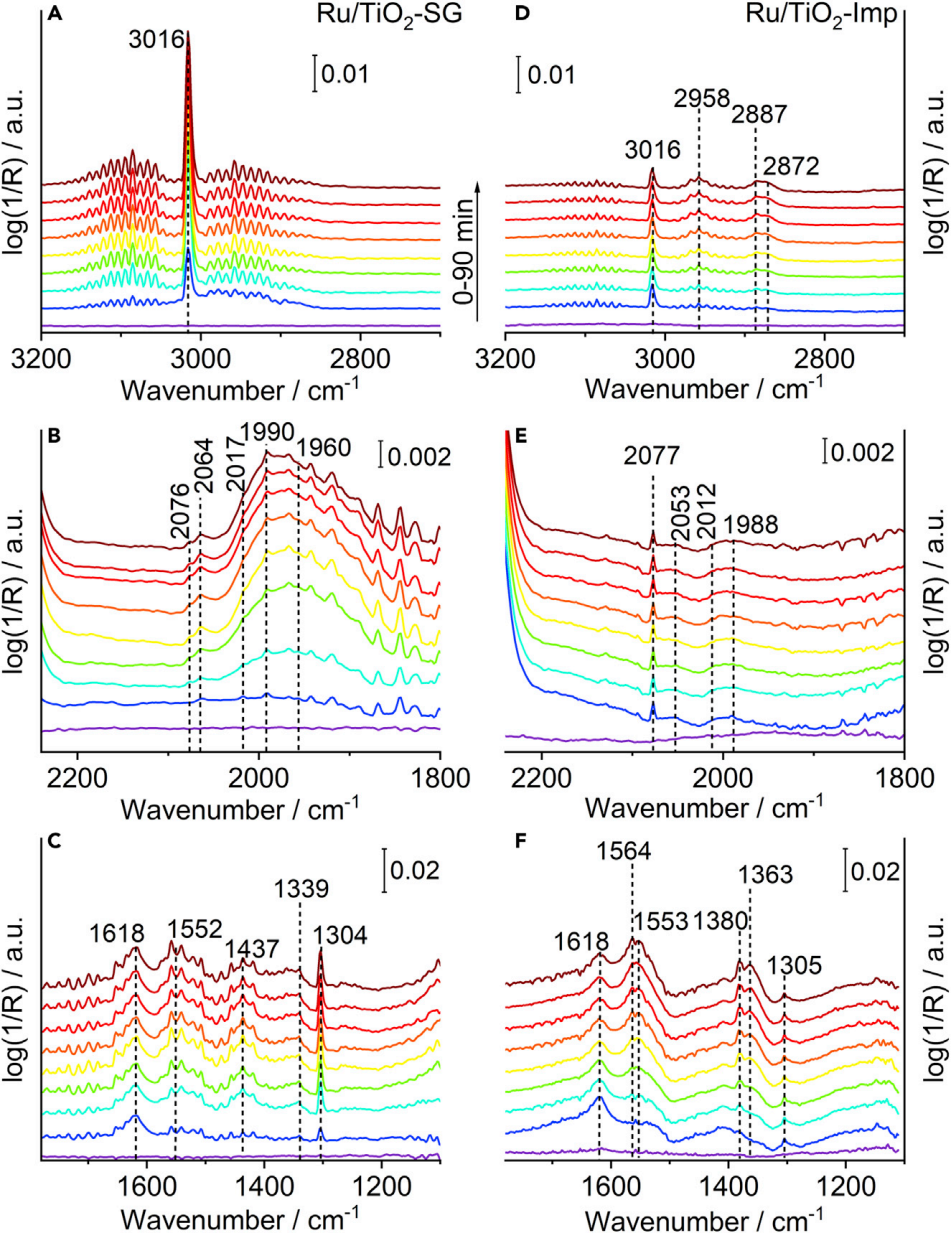
Operando DRIFTS measurements at 150°C (A and D) Spectra in the C-H region for Ru/TiO_2_-SG (A) and Ru/TiO_2_-Imp (D). (B and E) Spectra in the C-O region for Ru/TiO_2_-SG (B) and Ru/TiO_2_-Imp (E). (C and F) Spectra in the O-C-O region for Ru/TiO_2_-SG (C) and Ru/TiO_2_-Imp (F). From bottom to top: 0–90 min H_2_:CO_2_ = 4:1 (22.4 mL min^−1^ H_2_, 5.6 mL min^−1^ CO_2_, 2 mL min^−1^ He).

The formation of surface adsorbates and intermediates during the catalytic reaction was examined by operando DRIFT spectroscopy at 150°C (Figure 5). It is evident that the band intensity of gaseous CH_4_ at 3016 cm^−1^ (Gupta et al., 1994) was always much higher on Ru/TiO_2_-SG than on Ru/TiO_2_-Imp (Figures 5A and 5D), in line with the higher methane formation rate for the former catalyst. This is also confirmed by steady state spectra recorded in the temperature range up to 300°C for both catalysts together with the corresponding mass spectrometric analysis of the product flow leaving the DRIFT cell (Figure S5). Further bands are seen for catalyst Ru/TiO_2_-Imp from the combination of C-H bending with asymmetric (2958 cm^−1^) and symmetric O-C-O stretching vibrations (2887 cm^−1^), from C-H stretching (2872 cm^−1^), as well as from modes of formate species (Figure 5D) (Zhao et al., 2018). In the spectra of Ru/TiO_2_-SG, such bands might be obscured by the strong CH_4_ bands in (Figure 5A). The spectra of Ru/TiO_2_-SG in the C-O range are dominated by a very broad feature between 2050 and 1870 cm^−1^ that arises from the superposition of CO adsorbed on different Ru sites (Figure 5B). In literature, bands around 1960 cm^−1^ have been related to terminal CO adsorbed on Ru sites at the metal support interface (Panagiotopoulou, 2018; Panagiotopoulou et al., 2012), whereas bands around 1990 cm^−1^ were attributed to monodentate CO species on Ru sites of different oxidation states, including reduced Ru sites (Kamble et al., 1996; Kellner and Bell, 1981; Panagiotopoulou, 2017; Panagiotopoulou and Verykios, 2017). The weak feature at 2076 cm^−1^ may be assigned to Ru sites at the perimeter interface of Ru clusters (also suggested by EDS images discussed below) (Yan et al., 2018), whereas the weak band at 2064 cm^−1^ could be because of CO adsorbed at Ru^0^ species on terrace sites. Usually, such a signal appears below 2060 cm^−1^ (Gupta et al., 1994; Londhe et al., 1997; Loveless et al., 2013) but its position depends on the coverage. With increasing CO coverage, the Ru-CO bond weakens, leading to a strengthening of the C=O bond (Robbins, 1989). This may also explain the blue shift of the C=O band on Ru/TiO_2_-SG to 2064 cm^−1^. However, the most obvious difference between Ru/TiO_2_-SG and Ru/TiO_2_-Imp is the fact that the latter shows negligible band intensity in the C-O range around 1998 cm^−1^, indicating a much lower ability of the Ru sites to adsorb CO and/or a smaller number of such species accessible for reactants/intermediates.

In the region between 1200 and 1700 cm^−1^ (Figures 5C and 5F), the band at 1304–1305 cm^−1^ belongs to CH_4_, which is more intense in Ru/TiO_2_-SG, in line with its higher CH_4_ formation rate. The band at 1618 cm^−1^ might be associated with bidentate bicarbonate species (Pokrovski et al., 2001). Bands at 1363 and 1553 cm^−1^ stem from symmetric and asymmetric ν(O-C-O) vibrations of formate species (Marwood et al., 1997; Zhao et al., 2018). They appear with higher intensity on sample Ru/TiO_2_-Imp, on which also a band at 1380 cm^−1^ from C-H bending vibrations of formates is evident (Marwood et al., 1997; Zhao et al., 2018). This band is missing on Ru/TiO_2_-SG. This indicates that formate species, known to serve as source for the CO being itself the intermediate for CH_4_ formation (Wang et al., 2016b), are more stable on the less active Ru/TiO_2_-Imp catalyst while they quickly react further on Ru/TiO_2_-SG, being in line with its higher CH_4_ formation rate. Apart from the above discussed bands, there are weak features in Ru/TiO_2_-SG at 1437 cm^−1^ from bicarbonate species (Chen et al., 2016; Gupta et al., 1994) as well as at 1552 and 1339 cm^−1^ that likely stem from asymmetric and symmetric ν(O-C-O) vibrations of bidentate carbonate species (Yan et al., 2019). They may be located as spectators on the TiO_2_-SG support. On the other hand, they could also have formed after CO_2_ adsorption on O-vacancies in the vicinity of active Ru species, from where they are finally hydrogenated. On sample Ru/TiO_2_-Imp, the band at 1339 cm^−1^ is less pronounced, possibly because of the lower concentration of O-vacancies.

**Figure 6:**
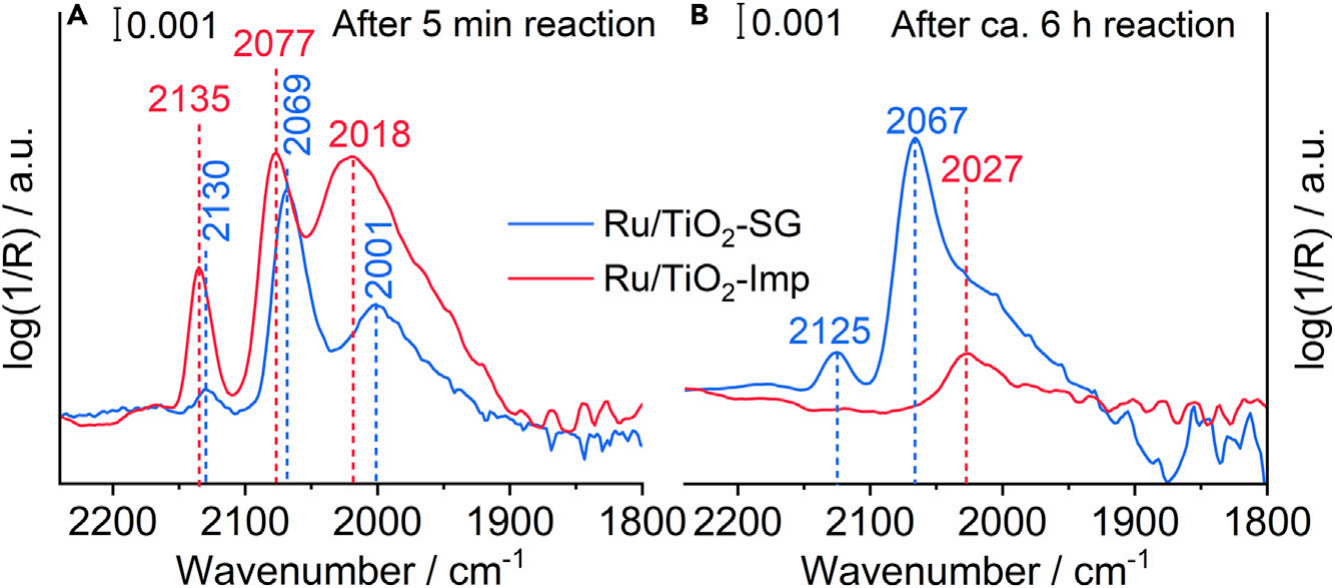
*In situ* DRIFTS measurements of CO adsorption at 30°C on spent catalysts after different reaction times (A) DRIFT spectra of the spent catalysts after a 5 min reaction. (B) DRIFT spectra of the spent catalysts after a 6 h reaction.

To obtain more information about the adsorption properties and the nature of the Ru surface species on the two catalysts, we performed CO adsorption at 30°C after exposing them to reaction conditions at 150°C for 5 min and 6 h, respectively. The spectrum of the Ru/TiO_2_-SG after 5 min reaction (Figure 6A), shows a band at 2130 cm^−1^ related to geminal CO adsorbed on low coordination Ru atoms (Loveless et al., 2013). The band at 2069 cm^−1^ may result from CO adsorbed on high coordination Ru^0^ sites which is blue-shifted because of higher CO coverage with respect to the situation at reaction conditions (Figure 5) (Robbins, 1989). Alternatively, the signal ca. 2069 cm^−1^ might be assigned to CO linearly adsorbed on Ru sites located at the periphery interface (Yan et al., 2018), which might be electronically modified by close interaction with the support (Chen et al., 2021). Finally, the band at 2001 cm^−1^ is typical for carbonyl groups adsorbed on top of Ru^0^ clusters (Solymosi and Raskó, 1989). In principle, the same bands occur also for the Ru/TiO_2_-Imp catalyst, yet they are all shifted to higher wavenumbers (Figure 6A). This indicates that the C=O bond on the latter catalyst is stronger than on Ru/TiO_2_-SG. The reason may be a more effective electron transfer from oxygen vacancies to Ru in the SG catalyst, as suggested by the *in situ* EPR results. This might promote backdonation of electron density from occupied d orbitals of Ru to antibonding 2π∗ orbitals of adsorbed CO, which weakens the C=O bond in the SG catalysts. After 6 h time on stream (Figure 6B), i.e., after completing the activation period and reaching the steady state (*cf.* Figure 2) this shift to lower wavenumbers is even more pronounced in the most active Ru/TiO_2_-SG catalyst, confirming the above discussed electron transfer. Remarkably, almost no CO is adsorbed on the less active Ru/TiO_2_-Imp catalyst after 6h on stream. This may be because of a partial diffusion of exposed Ru species into subsurface layers where they are not accessible anymore for CO (also suggested by a lower Ru:Ti surface ratio, see NAP-XPS measurements below) and to a partial increase of the Ru particle size (see HAADF-STEM and elemental mapping below) that causes a decrease in the population of sites in the periphery interface.

### NAP-XPS measurements

**Figure 7:**
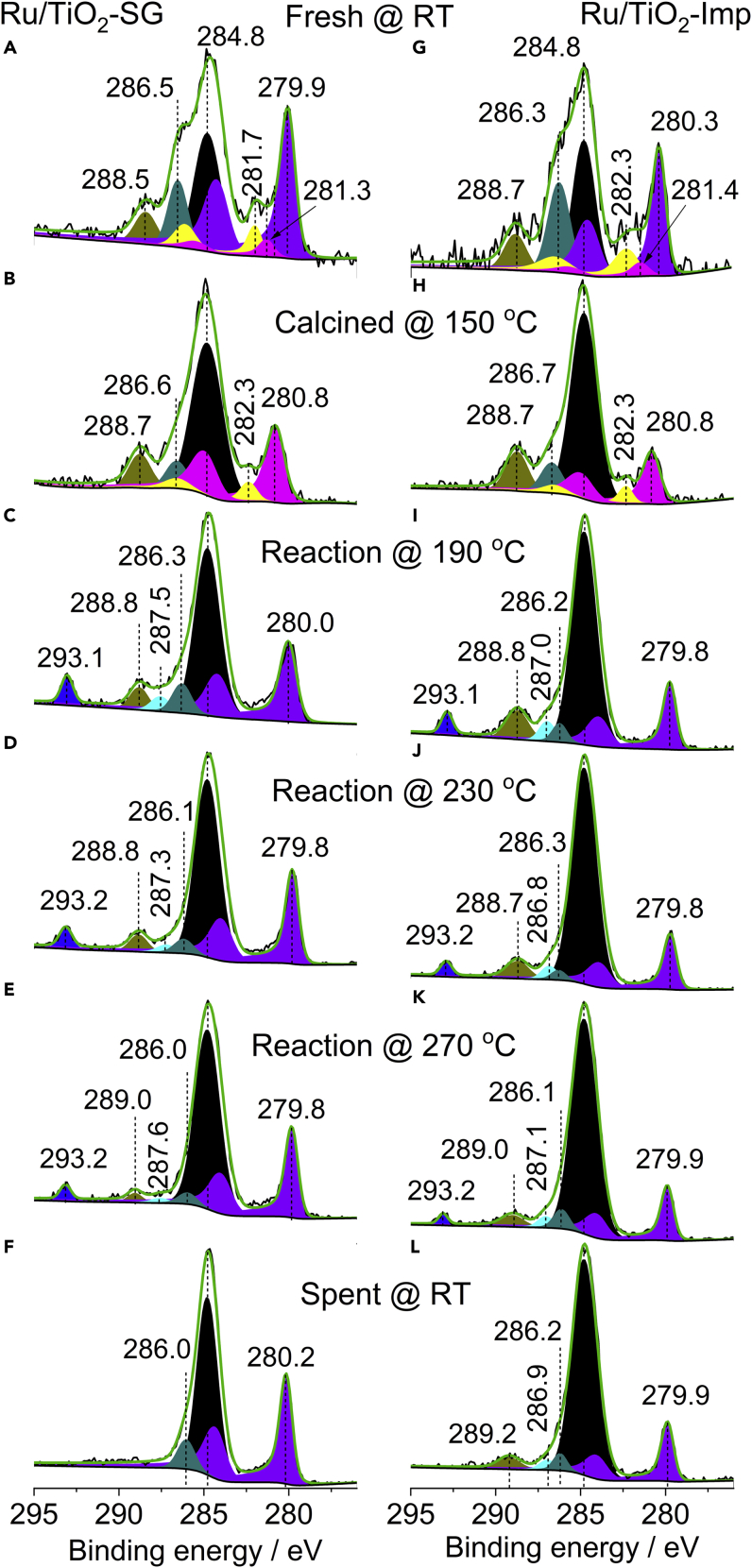
NAP-XPS spectra in the Ru 3days and C 1s region at different conditions. Experimental (black line) and fitted (green line) NAP-XPS spectra at a total pressure of 2 mbar, including deconvoluted subsignals (A and G) Fresh catalysts at RT in He. (B and H) Pre-oxidation at 150°C in 5 vol % O_2_/He. (C–K) Reaction in H_2_:CO_2_ = 4:1 at different temperatures. (F and L) Spent catalysts at RT in He.

The XP spectra recorded at different conditions are shown in Figure 7. The fresh catalysts without any pretreatment (Figures 7A and 7G), show predominantly metallic Ru^0^ species (3d_5/2_ peaks at 279.9 in Ru/TiO_2_-SG and 280.3 in Ru/TiO_2_-Imp) (Bianchi et al., 1991; Kim and Winograd, 1974). Small signals of Ru^4+^ at ca. 281.3 eV (Ru/TiO_2_-SG) and 281.4 eV (Ru/TiO_2_-imp) (Morgan, 2015), as well as satellite peaks of Ru 3d_5/2_ at 281.7 eV (Ru/TiO_2_-SG) and 282.3 eV (Ru/TiO_2_-Imp) were also resolved (Gu et al., 2016; Kim et al., 1997). The signals at 288.5 eV (Ru/TiO_2_-SG) and 288.7 eV (Ru/TiO_2_-Imp) together with the shoulders at 286.5 eV (Ru/TiO_2_-SG) and 286.3 eV (Ru/TiO_2_-Imp) can be attributed to organic surface deposits with C-O and O=C-O moieties (Greczynski and Hultman, 2020), whereas the C 1s signal at 284.8 eV from adventitious carbon was used as reference for all binding energies. After ca. 30 min oxidative pretreatment at 150°C in a flow of 5 vol % O_2_/He, the Ru^0^ peak disappeared while a Ru^4+^ signal at 280.8 eV grew on both catalysts (Figures 7B and 7H). This indicates oxidation of Ru^0^ to RuO_2_. Still, a contribution from the Ru 3d_5/2_ satellite at 282.3 eV is observed.

Upon switching to the reaction mixture (H_2_:CO_2_ = 4:1) at 190°C, this RuO_2_ was reduced again, as reflected by strong peaks at 280.8 eV for Ru/TiO_2_-SG (Figure 7C) and 279.8 eV for Ru/TiO_2_-Imp (Figure 7I). Moreover, two new C1s signals can be distinguished. The one at 293.1 eV is from gaseous CO_2_ (Kattel et al., 2016), whereas the peaks at 287.5 eV (Ru/TiO_2_-SG) and 287.0 eV (Ru/TiO_2_-Imp) lie in the range of formate-like species (Deng et al., 2008) as detected, too, in the DRIFT spectra. The signal at ca. 288.7–288.8 eV, on the other hand, lies in the binding energy range associated with carbonates (Deng et al., 2008). In principle, no significant changes were detected upon raising the reaction temperature stepwise to 270°C. In particular, no methoxy/methanol species were detected as it was the case in other reports (Kattel et al., 2016; Wang et al., 2016a). The respective mass related spectra recorded at the outlet of the spectrometer for different reaction conditions (190–270°C) are shown in Figure S8.

After stopping the CO_2_/H_2_ supply and cooling to room temperature, the formate signals disappeared on Ru/TiO_2_-SG (Figure 7F) while they were still visible on Ru/TiO_2_-Imp (Figure 7L). This indicates, in agreement with the DRIFTS results discussed above, that these species are less stable on the surface of the more active Ru/TiO_2_-SG catalyst. It is also in accordance with proposals from other studies regarding the role of these compounds as intermediates for the reduction of CO_2_ at similar conditions (Falbo et al., 2019; Wang et al., 2017; Zhao et al., 2018).

The NAP-XPS spectra in the Ti 2P region are dominated by the Ti 2p_3/2_ and 2p_1/2_ peaks at 458.7–458.9 and 464.4–464.5 eV typical for Ti^4+^ on both catalysts (Figure S6) (Biesinger et al., 2010). Reduced Ti^3+^ has not been detected for any of the two samples, in contrast to the EPR spectra (*cf.* Figure 4). However, this is no contradiction, considering that XPS is only sensitive to surface Ti^3+^ ions, whereas EPR detects all Ti^3+^ species in the sample, including those located in the bulk and the subsurface. Moreover, EPR is a very sensitive method that can detect even traces of Ti^3+^. If there are only very few Ti^3+^ species located on the surface, they might escape detection by XPS. An additional small signal is also resolved at 461.1–461.4 eV (Figure S7). This is the Ru 3p_3/2_ peak of metallic Ru^0^ (Qadir et al., 2012). It is only observed in the fresh catalysts and after treatment in CO_2_/H_2_ gas, but not after oxidative pretreatment at 150°C in 5 vol % O_2_/He, which is in agreement with the signals observed in the Ru 3d region (Figure 7).

**Figure 8:**
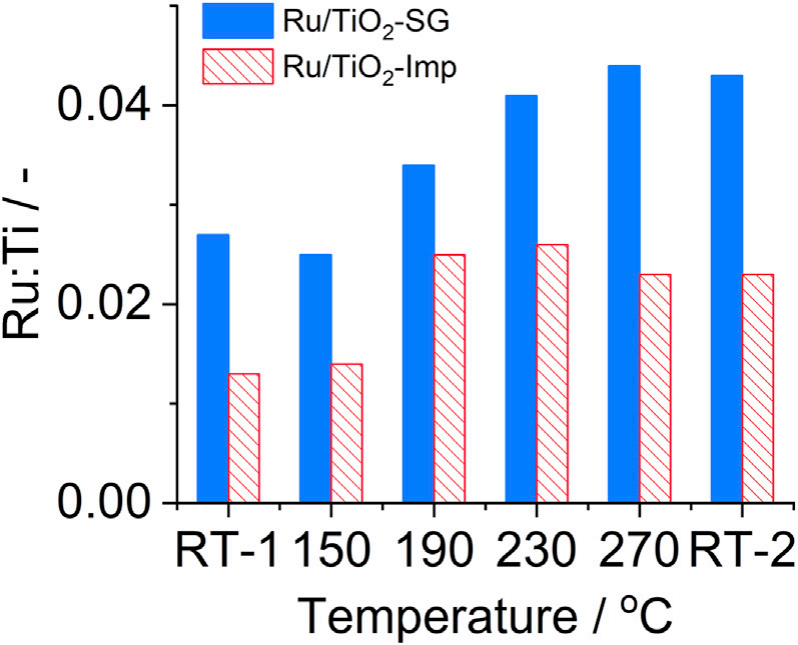
Operando NAP-XPS measurements: Ru:Ti atomic ratio Ru:Ti atomic ratio for the fresh catalysts at RT (RT-1), during calcination at 150°C, during reaction at 190, 230, 270°C, and after reaction at RT (RT-2).

The surface Ru/Ti ratios (Ru = total Ru) derived from the NAP-XP spectra under different conditions were always higher for the Ru/TiO_2_-SG catalyst (Figure 8). This points to a higher percentage of Ru surface species accessible to reactants which seems to increase even more with rising reaction temperature and might be one reason for the higher activity of the SG catalyst compared to Ru/TiO_2_-Imp (*cf.* Figure 2) (Abdel-Mageed et al., 2020, 2021; Carenco et al., 2016; Li et al., 2020). In contrast, the Ru/Ti ratio on the latter seems to decrease slightly with rising reaction temperature (Figure 8). Apart from temperature, the rising number of accessible Ru surface sites could have boosted CO_2_ conversion in catalyst Ru/TiO_2_-SG compared to sample Ru/TiO_2_-Imp (*cf.* Figure 3A).

### HAADF-STEM and elemental mapping

**Figure 9:**
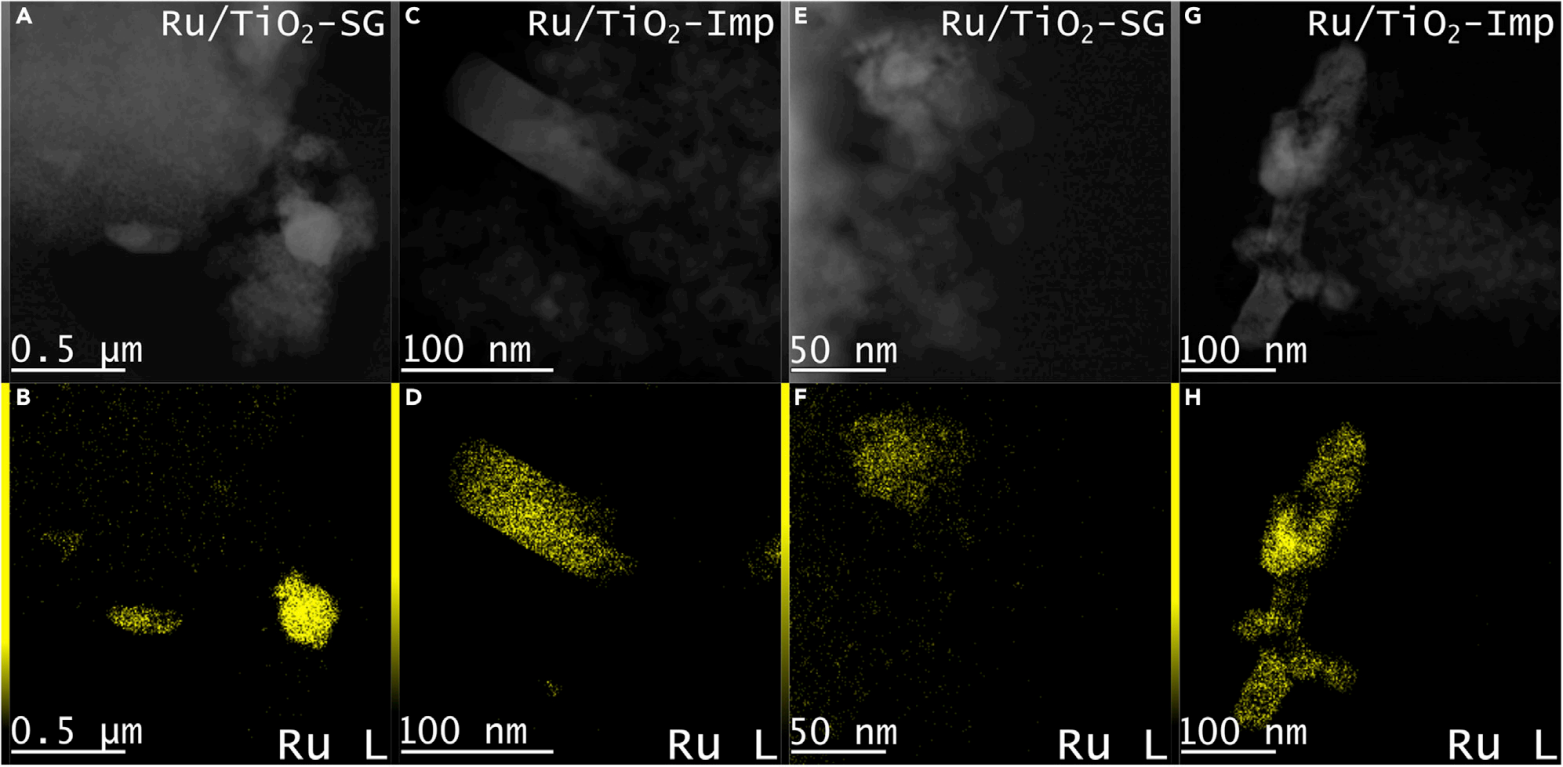
HAADF-STEM and elemental mapping (A and C) HAADF-STEM images of the fresh catalysts: Ru/TiO_2_-SG (A) and Ru/TiO_2_-Imp (C). (B and D) EDS maps of the Ru L signal of the fresh catalsts: Ru/TiO_2_-SG (B) and Ru/TiO_2_-Imp (D). (E and G) HAADF-STEM images of the spent catalysts: Ru/TiO_2_-SG (E) and Ru/TiO_2_-Imp (G). (F and H) EDS maps of Ru L signal of the spent catalysts: Ru/TiO_2_-SG (F) and Ru/TiO_2_-Imp (H). Micrographs of the spent catalysts were recorded after a 6 h reaction up to 270°C in H_2_:CO_2_ = 4:1.

HAADF-STEM and Ru EDS analysis of the as-prepared and spent Ru/TiO_2_-SG and Ru/TiO_2_-Imp catalysts are shown in Figure 9.

Both fresh catalysts contain few very large not uniformly distributed Ru particles (Figures S9 and S10). The major difference between them is that the more active catalyst Ru/TiO_2_-SG contains in addition many small highly dispersed Ru species (Figure 9B), possibly also single atoms, which are not present in sample Ru/TiO_2_-Imp (Figure 9D). After use in the catalytic reaction, the big particles in spent Ru/TiO_2_-SG are partly dissolved into smaller ones while the multitude of the tiny highly dispersed Ru species remained, in contrast to Ru/TiO_2_-Imp (Figures 9F and 9H). These highly dispersed Ru entities are partly composed of small subunits (see also Figures S11A, S11B, and S12). It is probable that the dissolution of big Ru particles also raised the number of Ru species accessible on the surface (reflected by the growing Ru/Ti ratio in NAP-XPS, Figure 8). In contrast, rather a growth than a partial dissolution of the big Ru particles was observed after reaction for Ru/TiO_2_-Imp (plot H, see also Figures S11C and S11D). This is in accordance with the slightly decreasing surface Ru/Ti ratio (Figure 8).

In addition, the presence of rutile TiO_2_ in Ru/TiO_2_-SG implies a possible strong interaction of TiO_2_ with Ru, which is not or only of minor relevance in Ru/TiO_2_-Imp.

### Structure-reactivity relationships

**Scheme 1:**
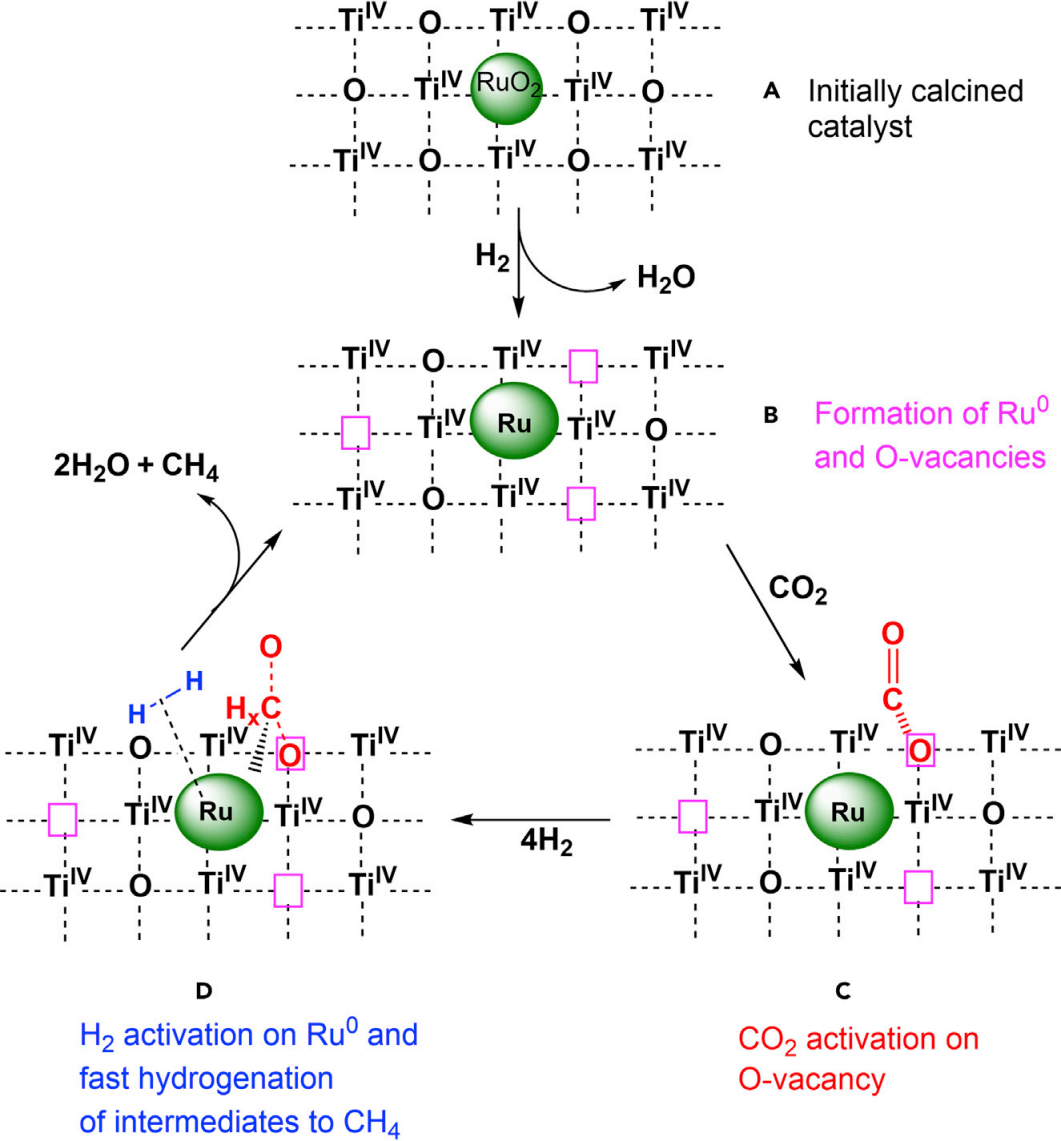
Structure-reactivity relationships Formation of O-vacancies and metallic Ru (A→B) and their role for activation of CO_2_ (C) and H_2_ (D).

Based on the results described in the previous sections, we propose the following structure-reactivity correlations for the highly active and selective Ru-TiO_2_-SG methanation catalyst. After oxidative pretreatment with 5 vol % O_2_ in He at 150°C, the catalyst contains oxidic Ru particles of ca. 3–4 nm mean diameter (confirmed by NAP-XPS and HAADF-STEM) and a negligible number of O-vacancies in the support (evident from *in situ* EPR, Scheme 1A). In the initial stage of the reaction, H_2_ reduces RuO_2_ to metallic Ru and removes lattice oxygen from the support to create O-vacancies. Because no Ti^3+^ ions were detected by *in situ* EPR and NAP-XPS, the electrons released by the removed O^2−^ species are most probably trapped by interfacial Ru sites instead of Ti^4+^, thus forming catalytically active Ru^0^ atoms at the metal/support interface on which molecular hydrogen is activated. Subsequently, CO_2_ adsorbs with one of its O atoms in the oxygen vacancy (Scheme 1C). This process weakens the C-O bond and makes it prone for facile hydrogenation by activated H species (Scheme 1D). Evidence for intermediates of this hydrogenation has been provided by bands in the carbonyl/formate region during operando DRIFTS measurements as well as by NAP-XPS measurements.

Consequently, we consider the formation of moieties containing tiny Ru species and O-vacancies in close vicinity as drivers for the extraordinary activity obtained with our Ru/TiO_2-_SG catalyst in low-temperature CO_2_ methanation. A dual role of O-vacancies is proposed for this reaction: 1) In the initial state of their formation, the electrons released by removal of oxygen atoms are quickly transferred to Ru^n+^ species at the metal/support interface, forming Ru^0^ sites for H_2_ activation. 2) The resulting empty O-vacancy acts as adsorption and activation site for CO_2_ (Lin et al., 2017; Nie et al., 2018; Sakpal and Lefferts, 2018).

### Conclusions

In this work, we have used an anhydrous sol-gel synthesis to develop a Ru catalyst with only 0.90 wt % Ru supported on a mesoporous TiO_2_ matrix that showed 100% selectivity for methanation of CO_2_ and much higher Ru-mass normalized reaction rates and TOF values than state-of-the-art Ru/TiO_2_ catalysts with higher Ru contents and the corresponding control catalyst prepared by conventional impregnation with the same Ru content. By dedicated sol-gel synthesis, it is possible to boost activity and selectivity of Ru/TiO_2_ catalysts in CO_2_ methanation well beyond the state of the art, while significantly saving precious ruthenium. To the best of our knowledge the reported methanation rates for the Ru/TiO_2_-SG catalyst below 200°C represent unprecedented high values for low-temperature CO_2_ methanation. The turnover frequency (TOF) was found to be at least 400% higher than that reported for other Ru/TiO_2_ catalysts under similar conditions. This much superior methane activity is probably driven by the creation of special O-vacancy-Ru^0^ sites at the metal-support perimeter interface which is obviously promoted by the sol-gel synthesis method, in contrast to catalysts prepared by other conventional methods that were much less active despite a significantly higher Ru content. This corroborates the suitability of our sol-gel methodology to create unique electronic interactions at the Ru-support interface required for maximizing the catalytic performance.

### Limitations of the study

By using a combination of several advanced *in situ* and operando spectroscopies, we gained strong indications that special Ru-*O*-vacancy sites created by a dedicated sol-gel synthesis route are the drivers of outstanding reaction rates in low-temperature methanation of CO_2_. Nevertheless, we feel that still more information, including theoretical analysis, is needed to identify precisely their immediate environment and the manner in which they participate in the formation of intermediates and in the total reaction mechanism. This may be achieved by using X-ray absorption methods and DFT calculations. Such assessments/calculations, however, have not been performed.

## Supporting citations

The following references appear in the supplemental information: Abdel-Mageed et al., 2020, 2021; Abe et al., 2009; Chai et al., 2019; Kim et al., 2018; Kim et al., 2016; Lin et al., 2014, 2017; Petala and Panagiotopoulou, 2018; Sassoye et al., 2011.

## STAR★Methods

### Key resources table

**Table:** 

REAGENT or RESOURCE	SOURCE	IDENTIFIER
**Chemicals, peptides, and recombinant proteins**		

Ti(IV) Isopropoxide	Sigma-Aldrich	Cat#377996
Ethyl acetoacetate	Sigma-Aldrich	Cat#537349
RuCl_3_•3H_2_O	Sigma-Aldrich	Cat#10452

**Other**		

Nicolet 6700 FTIR spectrometer	Thermo Scientific	https://www.thermofisher.com/search/results?query=FTIR&focusarea=Suchen%20in%20Alle
ELEXSYS 500-10/12 X-band cw EPR spectrometer	Bruker	https://www.bruker.com/en/products-and-solutions/mr/epr-instruments/epr-research-instruments/Elexsys-II-E500-CW-EPR.html
NAP-XPS spectrometer	SPECS Surface Nano Analysis GmbH	https://www.specs-group.com/nc/specs/products/detail/nap-xps-system-exchangeable-chambers/

### Resource availability

#### Lead contact

Further information and requests for resources should be directed to and will be fulfilled by the lead contact, Dr. Jabor Rabeah (Jabor.Rabeah@catalysis.de).

#### Materials availability

All materials generated in this study will be made available upon reasonable request to the lead contact. All the chemical reagents and synthesis procedures are summarized below.

### Method details

#### Support and catalyst preparation

The TiO_2_ support was prepared by a sol-gel method. Ti(IV) isopropoxide (3.75 mL) and ethyl acetoacetate (EAcAc) (1.62 mL) were mixed and magnetically stirred at room temperature for 1 h. Afterwards HNO_3_ (2.25 ml of 0.1 M) was dropwise added at room temperature under continuous stirring to form a transparent gel by hydrolysis. The gel was subsequently extracted by supercritical ethanol (30 ml) in an autoclave (T = 245°C, p = 60 bar) during 10 min to create an aerogel which was then calcined at 500°C for 3 h in static air. For the synthesis of the Ru/TiO_2_ sol-gel catalyst (Ru/TiO_2_-SG), RuCl_3_⋅H_2_O (0.16 g) solved in anhydrous ethanol (8.75 ml) was also added to the mixture of Ti(IV) isopropoxide and ethyl acetoacetate. After this, the followed procedure was identical to that described for synthesizing the bare TiO_2_ support. A control catalyst was prepared by an incipient wetness impregnation method. RuCl_3_⋅H_2_O (0.018 g) dissolved in ultrapure water (13 ml) was homogeneously distributed on the surface of the sol-gel prepared TiO_2_ support (0.5 g) using a syringe. The impregnated sample was left for 4 days at RT and afterwards calcined in synthetic air at 500°C during 3 h. This catalyst is labelled as Ru/TiO_2_-Imp.

#### Catalyst characterization

##### Elemental analysis and specific surface area (SSA)

The elemental compositions were determined by inductively coupled plasma optical emission spectroscopy (ICP-OES) using a 715-ES ICP emissions spectrometer (Varian, Palo Alto, CA, USA). The samples were digested in a mixture of HF and aqua regia and then treated in a microwave-assisted sample preparation apparatus at 200°C and 60 bar.

BET surface area and pore volume were calculated from N_2_ adsorption isotherms measured at -196°C using a Micromeritics ASAP 2010 device. Before measurement, each sample was degassed at 200°C for 4 h. The average pore diameters were calculated from the desorption branch of the isotherm using the BJH method.

##### X-ray diffraction (XRD)

XRD powder patterns were recorded by an X’Pert Pro, Panalytical spectrometer equipped with a X’Celerator RTMS detector using Cu Kα radiation (λ = 0.154 nm) and a 2θ angle ranging from 5 to 80°.

##### Hydrogen temperature programmed reduction (H_2_-TPR)

H_2_-TPR measurements were performed in a Micromeritics Autochem II 2920 instrument. For assessing the H_2_ consumption on the support, 100 mg of bare TiO_2_ were loaded in U shaped quartz reactor. The H_2_-TPR run was carried out from RT to 800°C in a 5% H_2_/Ar flow (30 ml min^-1^) with a heating rate of 10 °C min^-1^. In the case of the Ru/TiO_2_ catalyst, 50 mg were loaded and heated from RT to 700°C with a heating rate of 20 °C min^-1^. The hydrogen consumption signals were recorded using a TCD detector.

##### Scanning transmission electron microscopy (STEM)

STEM measurements were performed with a probe aberration-corrected JEM-ARM200F (Jeol, Corrector: CEOS) at 200 kV. The microscope is equipped with a JED-2300 (JEOL) energy-dispersive x-ray-spectrometer (EDXS) including a silicon drift detector (dry SD60GV) for chemical analysis, and with an Enfinium ER (Gatan) electron energy loss spectrometer (EELS). A High-Angle Annular Dark Field (HAADF) and an Annular Bright Field (ABF) detector were used for STEM imaging, while EELS acquisition was performed using the Annular Dark Field (ADF) detector. Deposition of the solid samples was conducted without any pretreatment on a holey carbon supported Cu-grid (mesh 300), which was then transferred to the microscope.

##### Raman spectroscopy

Ex-situ Raman spectra were obtained at ambient temperature and pressure with a Horiba Jobin Yvon LabRam micro-spectrometer iHR 550 spectrometer using a 633 nm laser source. Spectra were acquired from different areas of the sample to check for sample homogeneity, using a laser power on the samples of 0.1 mW to 10 mW with a power density of 2.8 x 105 W∗cm^-2^. Data analysis was performed by LabSpec 6, Jobin Yvon Horiba, built-in software.

##### Catalyst preparation for in-situ/operando measurements

Prior to the characterization under reaction conditions, both, the TiO_2_ support as well as the Ru/TiO_2_ catalysts were calcined under 5 vol.% O_2_ (30 ml min^-1^) during 30 min at 150°C.

##### Operando diffuse reflectance infra-red Fourier transform spectra (DRIFTS)

DRIFT spectra were collected on a Nicolet 6700 FTIR spectrometer using a high-temperature Praying Mantis reaction cell (Harrick) with CaF_2_ windows equipped with a temperature control unit (Eurotherm) and connected to a gas dosing system with mass-flow controllers (Bronkhorst). Each spectrum was recorded with a resolution of 4 cm^-1^. 50 scans were averaged. 20 mg catalyst powder diluted with 60 mg α-Al_2_O_3_ (pre-calcined at 900°C in synthetic air for 8 h) were deposited over a layer of 80 mg pure α-Al_2_O_3_ within the sample cup. This dilution was necessary to reduce light absorption of the pure dark catalyst. The cell was flushed with 30 ml min^-1^ He after calcination while the temperature was increased to 300 °C at a rate of 15 °C min^-1^. Subsequently, the temperature was decreased at the same rate and background spectra were taken for each reaction temperature. The reaction was performed in a temperature range between 190 - 300°C under 4:1 H_2_:CO_2_ (22.4 ml min^-1^ H_2_, 5.6 ml min^-1^ CO_2_, 2 ml min^-1^ He). The intensity of the signals is given in log(1/R) scale. The gas outlet was connected to a quadrupole mass spectrometer (Omnistar, Pfeiffer Vacuum GmbH) for online product analysis.

##### CO adsorption at 30°C

After reaction and subsequent flushing at 150°C with He, the DRIFTS system was cooled down to 30°C in He atmosphere. Once the target temperature was reached, a gas mixture consisting of 5 vol.% CO/He (30 ml min^-1^, atmospheric pressure) was fed into the reaction cell for 1 h (until saturation) and CO adsorption was followed by recording DRIFT spectra during this time with the same resolution as used for operando-DRIFTS assessments described before.

##### Operando electron paramagnetic resonance (EPR)

Operando EPR spectra were recorded in a temperature range between 190 - 270°C, in a flow of 28.8 ml min^-1^ H_2_ + 7.2 ml min^-1^ CO_2_ + 4 ml min^-1^ Ar after pre-calcination in 40 ml min^-1^ 5 vol.% O_2_/He followed by He flushing (40 ml min^-1^) at 150°C. The spectra were recorded in an ELEXSYS 500-10/12 X-band cw spectrometer (Bruker) using a modulation frequency and amplitude of 100 kHz and up to 5 G, respectively. Typically, 100 mg of sample were loaded in a quartz plug-flow reactor connected to a gas dosing unit equipped with mass flow-controllers (Bronkhorst) at the inlet while the outlet gases were conducted to a quadrupole mass spectrometer (Omnistar, Pfeiffer Vacuum GmbH) for online product analysis.

##### Near ambient X-ray photoelectron spectroscopy (NAP-XPS)

Spectra were recorded on a laboratory NAP-XPS (SPECS Surface Nano Analysis GmbH, Germany). The setup is equipped with a differentially pumped Phoibos 150 electron energy analyser with a nozzle of 500 μm, a monochromated Al Kα radiation source (E = 1486.6 eV) and a laser heating system for sample heating. The analysis chamber was connected to three mass flow controllers (Brooks, GF40) for dosing reaction mixtures up to a total pressure of 2 mbar. Reaction gases (4:1 H_2_:CO_2_) and formed products were monitored by a quadrupole mass spectrometer (QMS, MKS e-vision 2) attached to the lens system of the spectrometer. The powder samples were pressed on a stainless-steel sample plate using a laboratory press with 5 mm diameter and a load of about 0.5 t. Temperature was monitored by a thermocouple on the sample plate pressed to the sample surface. The electron binding energies were referenced to the C 1s core level of carbon at 284.8 eV (C-C and C-H bonds). For analysis, the peaks were deconvoluted into Gaussian-Lorentzian curves using the software Unifit 2021.

#### Catalytic tests

The kinetic experiments were performed in a fixed-bed quartz tube micro-reactor (with 4 mm inner diameter) at atmospheric pressure under a total gas flow of 40 ml min^−1^ with a 4:1 H_2_:CO_2_ gas mixture (32 ml min^-1^ H_2_, 8 ml min^-1^ CO_2_). The catalyst was diluted in a ratio of 1:10 with inactive and thermally stable α-Al_2_O_3_ powder (calcined at 900 °C for 24 h) to ensure differential reaction conditions (conversion < 20%). In total, about 200 mg of the diluted catalyst was used during the measurements. After calcination, the catalyst was in-situ reduced after switching to the reaction mixture. The influent and effluent gases were analyzed by online gas chromatography with a CO detection limit of ca. 5 ppm (DANI 86.10), using thermal conductivity detectors (H_2_ used as carrier gas) and a standard test gas mixture for calibration. The Ru-mass-normalized reaction rate for CO_2_ methanation (CO_2_ + 4H_2_ ⇆ CH_4_ + 2H_2_O) was calculated from the CO_2_ conversion (X_CO2_) under differential reaction conditions (X_CO2_ < 20%), the molar flow rate of CO_2_ into the reactor (n_CO2,in_), and the absolute mass of Ru metal (m_Ru_) according to Equation 1. The Ru-mass-normalized CH_4_ formation rate, in contrast, was calculated from the effluent molar flow rate of the CH_4_ formed (n_CH4,out_), which was produced from CO_2_ (Equation 2). From these Ru-mass-normalized reaction rates, the selectivity to CH_4_ the selectivity for CO_2_ methanation (S_CH4(CO2)_) with respect to the reverse water-gas shift reaction (RWGS) is given by the ratio of the CO_2_ methanation rate compared to that of the overall CO_2_ conversion (methanation and RWGS, see Equation 3). The reported turnover frequencies (TOFs) were calculated by Equation 7 using the molar mass of Ru (M_Ru_) and the Ru dispersion (D_Ru_) obtained from TEM imaging according to Equations 4 and 6. In Equation 4 d_i_ is the estimated size of a Ru NP in nm while n_i_ is the percentage of particles with a size d_i_. Analogously, ν is the volume of an Ru atom (13.5 Å^3^) and s the surface area of an Ru atom (6.67 Å^2^).

(Equation 1) $$R_{{\text{CH}}_{4}}=\frac{{\text{n}}_{{\text{CH}}_{4},\text{out}}}{{\text{m}}_{\text{Ru}}}$$

(Equation 2) $$R_{{\text{CO}}_{2}}=\frac{{\text{X}}_{{\text{CO}}_{2}}{\text{n}}_{{\text{CO}}_{2},\text{in}}}{{\text{m}}_{\text{Ru}}}$$

(Equation 3) $$S_{{\text{CH}}_{4}\left(CO_{2}\right)}=\frac{R_{{\text{CH}}_{4}}}{R_{{\text{CO}}_{2}}}\;=\frac{R_{{\text{CH}}_{4}}}{R_{{\text{CH}}_{4}}+R_{CO}}$$

(Equation 4) $$d_{va}=\left(\frac{\sum_{i}n_{i}d_{i}^{3}}{\sum_{i}n_{i}d_{i}^{2}}\right)$$

(Equation 5) $$\text{TOF}=\left(\frac{C{H_{4}}_{molecules}}{N_{Ruatoms}*s}\right)$$

(Equation 6) $${\text{D}}_{\text{Ru}}=10\left(\frac{6v}{s}\right)\left(\frac{1}{d_{va}}\right)$$

(Equation 7) $$\text{TOF}=\frac{R_{{\text{CH}}_{4}}{\text{M}}_{\text{Ru}}}{{\text{D}}_{\text{Ru}}}=\left(\frac{moles_{CH4}}{g_{cat}*s}\right)/\left(\frac{moles_{Rusurface}}{g_{cat}}\right)$$

## Data Availability

• All data reported in this paper will be shared by the lead contact upon request. • This study does not report original code. • Any additional information required to reanalyze the data reported in this paper is available from the lead contact upon request.
