# Supplementary material for: Oxygen vacancies in Ru/TiO2 - drivers of low-temperature CO2 methanation assessed by multimodal operando spectroscopy
Source: iScience. 2022 Feb 8;25(3):103886. doi: 10.1016/j.isci.2022.103886 (PMC8861654; doi:10.1016/j.isci.2022.103886)
Supplement: Document S1. Figures S1–S13 and Table S1 [file mmc1.pdf]

iScience, Volume *25*

Supplemental information

Oxygen vacancies in Ru/TiO_2 - drivers of low-temperature CO_2 methanation assessed by multimodal operando spectroscopy

Sebastian Cisneros, Ali Abdel-Mageed, Jawaher Mosrati, Stephan Bartling, Nils Rockstroh, Hanan Atia, Hayder Abed, Jabor Rabeah, and Angelika Brückner

Supplemental Information

Table of contents

Supplemental figures

Figure S1: Raman spectra of the fresh materials.
Figure S2: N_2 physisorption isotherms and pore properties of the fresh materials.
Figure S3: H_2-TPR spectra of the bare TiO_2-SG support, Ru/TiO_2-SG and Ru/TiO_2-Imp catalysts.
Figure S4: In-situ EPR spectra of Ru/TiO_2-SG and Ru/TiO_2-Imp recorded at reaction conditions up to 270 ℃, H_2:CO_2 = 4:1.
Figure S5: Operando steady state DRIFT spectra and respective mass related spectra at the outlet of DRIFT cell during reaction on Ru/TiO_2-SG and Ru/TiO_2-Imp at different temperatures, 150 - 300 ℃, H_2:CO_2 = 4:1.
Figure S6: NAP-XPS spectra in the Ti2p region at different conditions.
Figure S7: NAP-XPS spectra in the Ru3p region at different conditions.
Figure S8: Mass related spectra at the outlet of XP spectrometer during reaction on Ru/TiO_2-SG and Ru/TiO_2-Imp at different temperatures, 190 - 270 ℃, H_2:CO_2 = 4:1.
Figure S9: STEM-HAADF image and selected EDX spectra of distinct areas of fresh Ru/TiO_2-SG.
Figure S10: STEM-HAADF image and selected EDX spectra of distinct areas of fresh Ru/TiO_2-Imp.
Figure S11: Selected HAADF-STEM micrographs of the spent catalysts after reaction up to 270 ℃ in H_2:CO_2 = 4:1.
Figure S12: Selected HAADF-STEM micrographs of Ru/TiO_2-SG.
Figure S13: Ru particle size distribution in the spent Ru/TiO_2-SG catalyst after reaction up to 270 ℃, H_2:CO_2 = 4:1.
Table S1. Reported activity of CO_2 methanation on different Ru/TiO_2 catalysts synthesized by different methods.

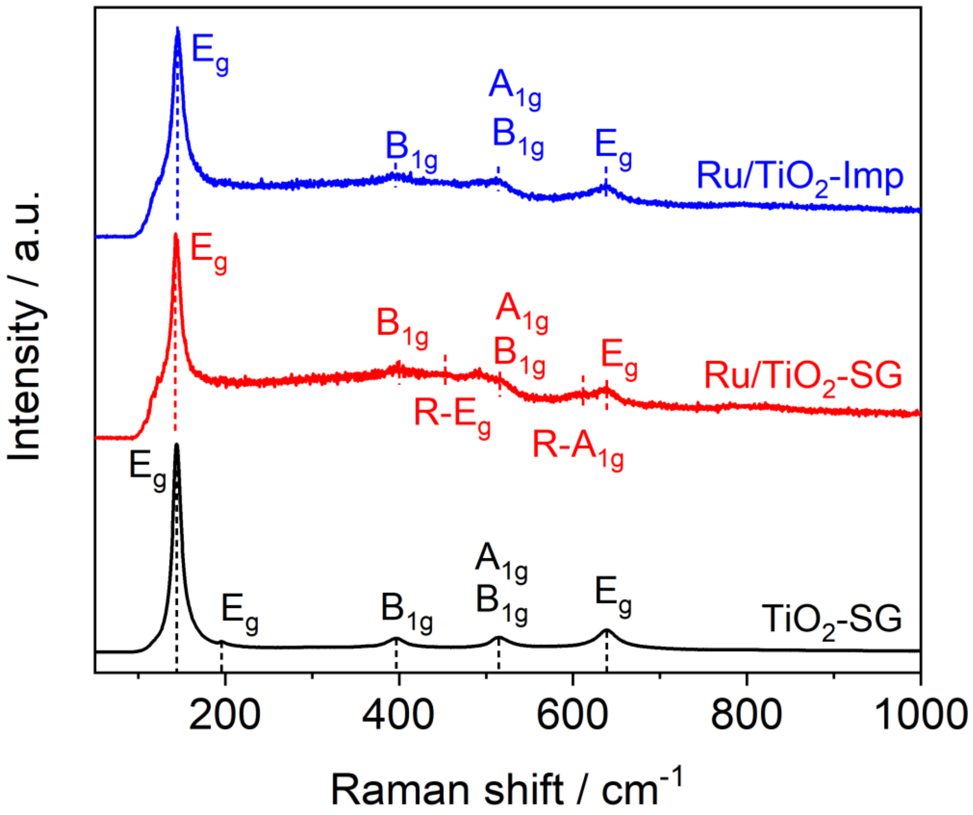

Figure S1. Raman spectra of the fresh materials. Related to STAR Methods. Weak R-E_g and R-A_{1g} signals in Ru/TiO_2-SG are not evident in the spectrum of the Ru/TiO_2-Imp catalyst. These signals might be related with planar O-O and Ti-O stretching in rutile. This is in agreement with the respective observations from XRD patterns shown in Figure 1. See experimental details in Method Details section.

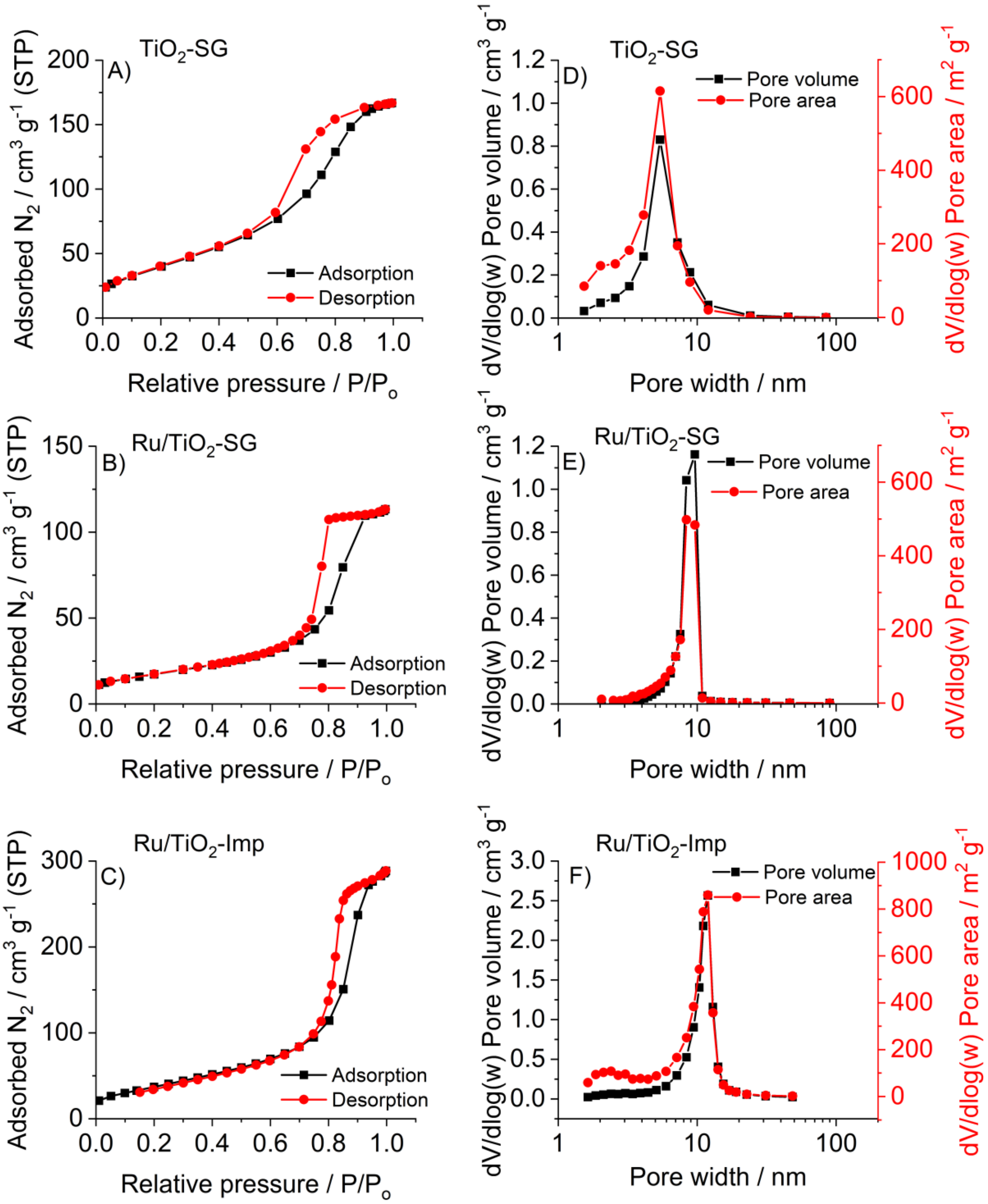

Figure S2. Pore properties of the fresh materials. Related to STAR Methods.

N_2 adsorption isotherms of fresh bare TiO_2 support (A), fresh Ru/TiO_2-SG catalyst (B), fresh Ru/TiO_2-Imp catalyst (C).

Pore volume and specific pore area of fresh bare TiO_2 support (D), fresh Ru/TiO_2-SG catalyst (E), fresh Ru/TiO_2-Imp catalyst (F).

The marked differences in BET SSA and pore volume between Ru/TiO_2-SG and Ru/TiO_2-Imp (see also Table 1) are not caused by the thermal pretreatment but might be due to special interactions of the Ru component with the support resulting from the preparation route. See experimental details in Method Details section.

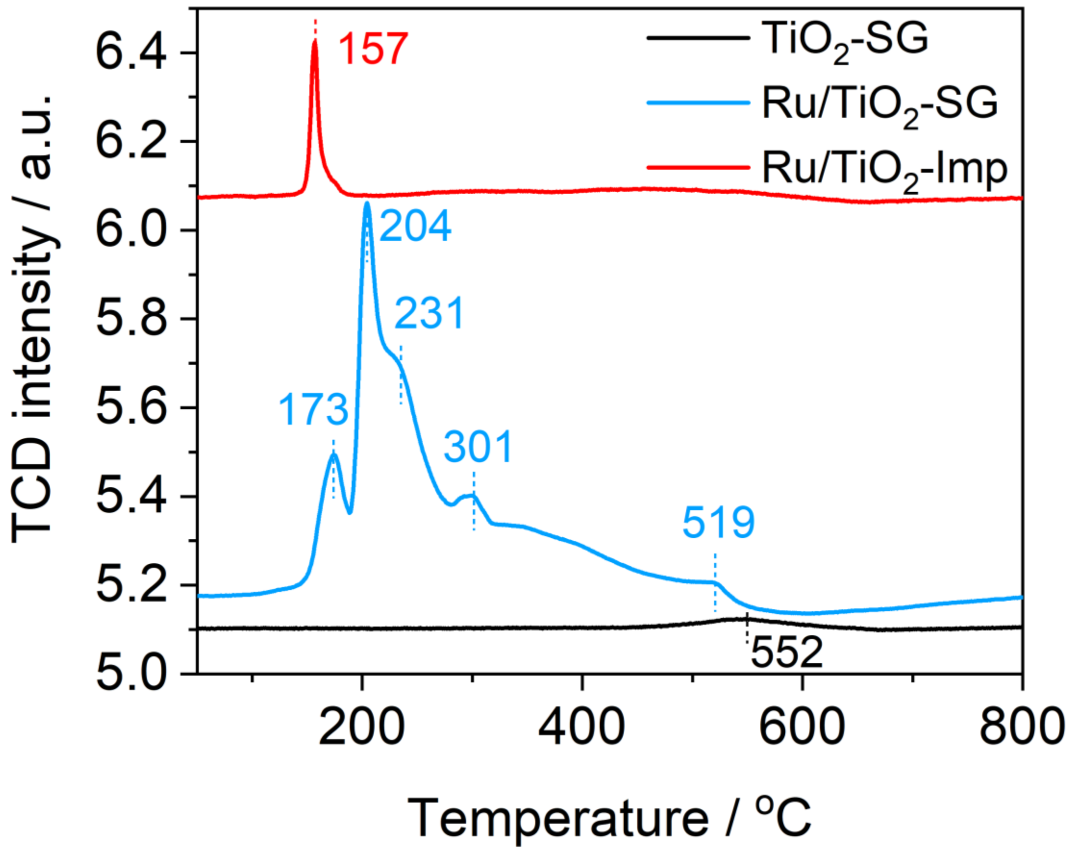

Figure S3. H_2 TPR spectra of the bare TiO_2-SG support, Ru/TiO_2-SG and Ru/TiO_2-Imp catalysts. Related to STAR Methods. Only a weak single peak at ca. 552 °C is observed for the bare support. The Ru/TiO_2-SG catalyst shows several H_2 consumption peaks between 170 and 520 °C, resulting from Ru species which differ in their oxidation states and/or the strength of their interaction with the carrier. In contrast, Ru/TiO_2-Imp shows only a narrow reduction peak at 157 °C and the amount of consumed H_2 is much lower, ca. 21 % of that observed for Ru/TiO_2-SG. The reason for these differences points to stronger metal-support interactions in the case of Ru/TiO_2-SG when compared to Ru/TiO_2-Imp. See experimental details in Method Details section.

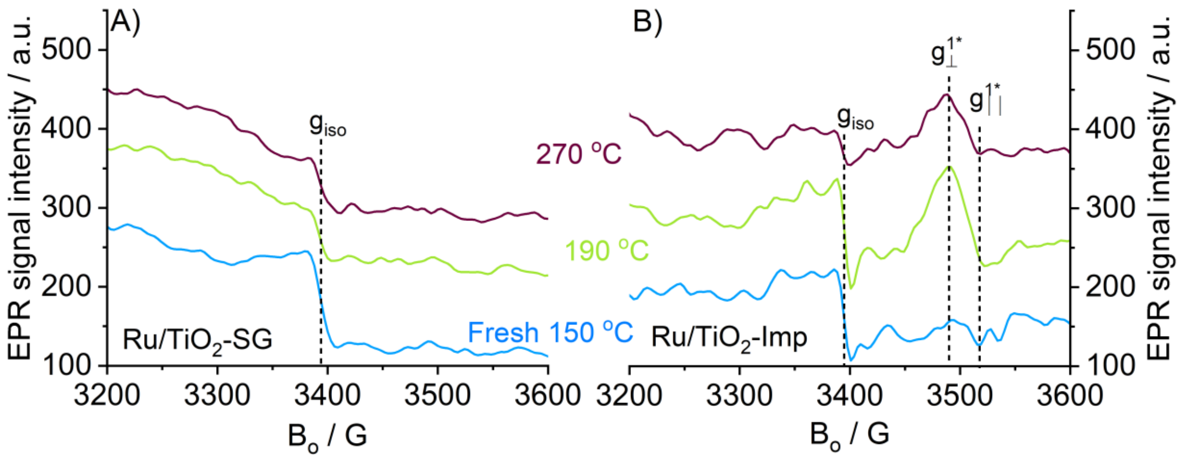

Figure S4. In-situ EPR measurements at different reaction conditions. Related to STAR Methods and complementary to Figure 4. See experimental details in Method Details section.
(A) Ru/TiO_2-SG fresh catalyst at 150 °C in He and at reaction conditions at 190 °C and 270 °C, H_2:CO_2 = 4:1 (28.8 ml min^{-1} H_2 + 7.2 ml min^{-1} CO_2 + 4 ml min^{-1} Ar).
(B) Ru/TiO_2-Imp fresh catalyst at 150 °C in He and at reaction conditions at 190 °C and 270 °C, H_2:CO_2 = 4:1 (28.8 ml min^{-1} H_2 + 7.2 ml min^{-1} CO_2 + 4 ml min^{-1} Ar).
The absence of signals related to Ti^{3+} in Ru/TiO_2-SG suggests that electrons released in the anion vacancies after O removal are transferred preferentially to Ru^{n+} species at the metal-support interface, reducing them to EPR-silent Ru species.

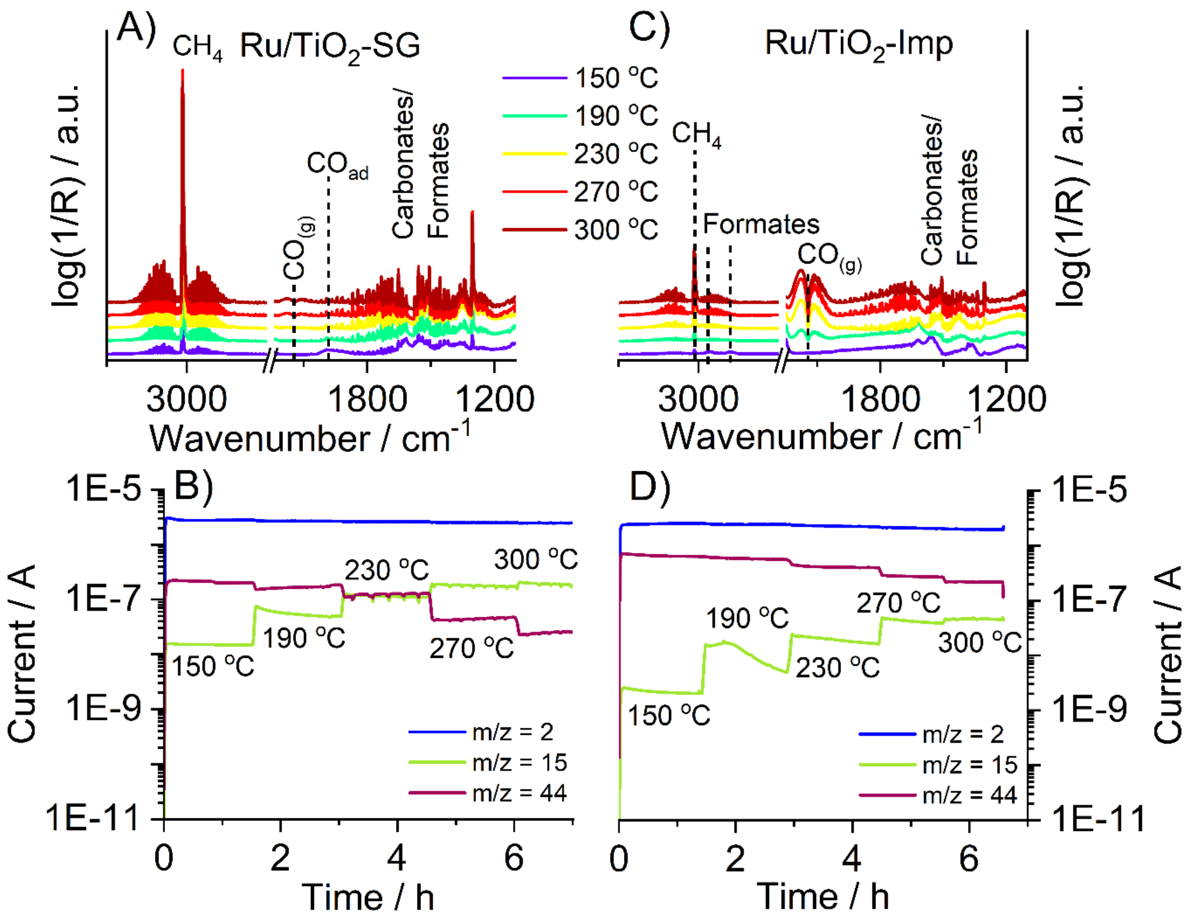

Figure S5. Operando steady state DRIFT spectra at different reaction conditions and corresponding mass spectra recorded during the measurement. Related to STAR Methods. See experimental details in Method Details section.

(A) DRIFT Spectra of Ru/TiO_2-SG catalyst from 150 °C to 300 °C, H_2:CO_2 = 4:1 (22.4 ml min^{-1} H_2 + 5.6 ml min^{-1} CO_2 + 2 ml min^{-1} He).

(B) Mass spectra of reaction related species (m/z = 2, H_2; m/z = 15, CH_3; m/z = 44, CO_2) at the outlet of the DRIFTS cell at different reaction conditions on Ru/TiO_2-SG: 150 °C to 300 °C, H_2:CO_2 = 4:1 (22.4 ml min^{-1} H_2 + 5.6 ml min^{-1} CO_2 + 2 ml min^{-1} He).

(C) DRIFT Spectra of Ru/TiO_2-Imp catalyst from 150 °C to 300 °C, H_2:CO_2 = 4:1 (22.4 ml min^{-1} H_2 + 5.6 ml min^{-1} CO_2 + 2 ml min^{-1} He).

(D) Mass spectra of reaction related species (m/z = 2, H_2; m/z = 15, CH_3; m/z = 44, CO_2) at the outlet of the DRIFTS cell at different reaction conditions on Ru/TiO_2-Imp: 150 °C to 300 °C, H_2:CO_2 = 4:1 (22.4 ml min^{-1} H_2 + 5.6 ml min^{-1} CO_2 + 2 ml min^{-1} He).

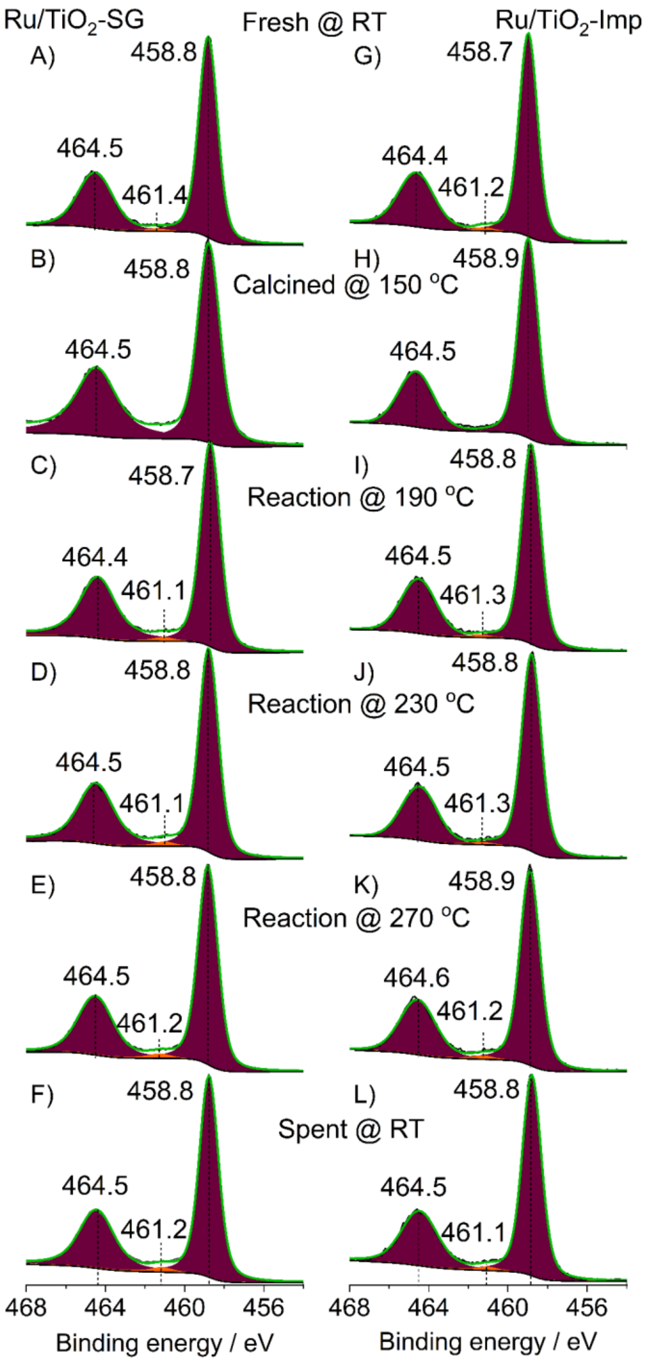

Figure S6. NAP-XPS spectra of Ru/TiO_2-SG and Ru/TiO_2-Imp in the Ti 2p region recorded at different conditions. Green lines denote the fit sum. Related to STAR Methods and complementary to Figure 7. See experimental details in Method Details section.

(A, G) Fresh catalysts at RT in He.

(B, H) Oxidation pretreatment before reaction at 150 ℃. 5 vol.% O_2/He.

(C-K) Catalyst at different temperatures. H_2 :CO_2 = 4:1.

(F, L) Spent catalysts at RT in He.

Since reduced Ti^{3+} has not been detected for any of the two samples, in contrast to the EPR spectra (see Figure 4), these observations demonstrate the sensitivity differences between both techniques. However, the additional small signal resolved at 461.1-461.4 eV corroborates that the oxidation state of Ru, after switching the atmosphere to reaction conditions, corresponds to metallic Ru as also observed in Figure 7.

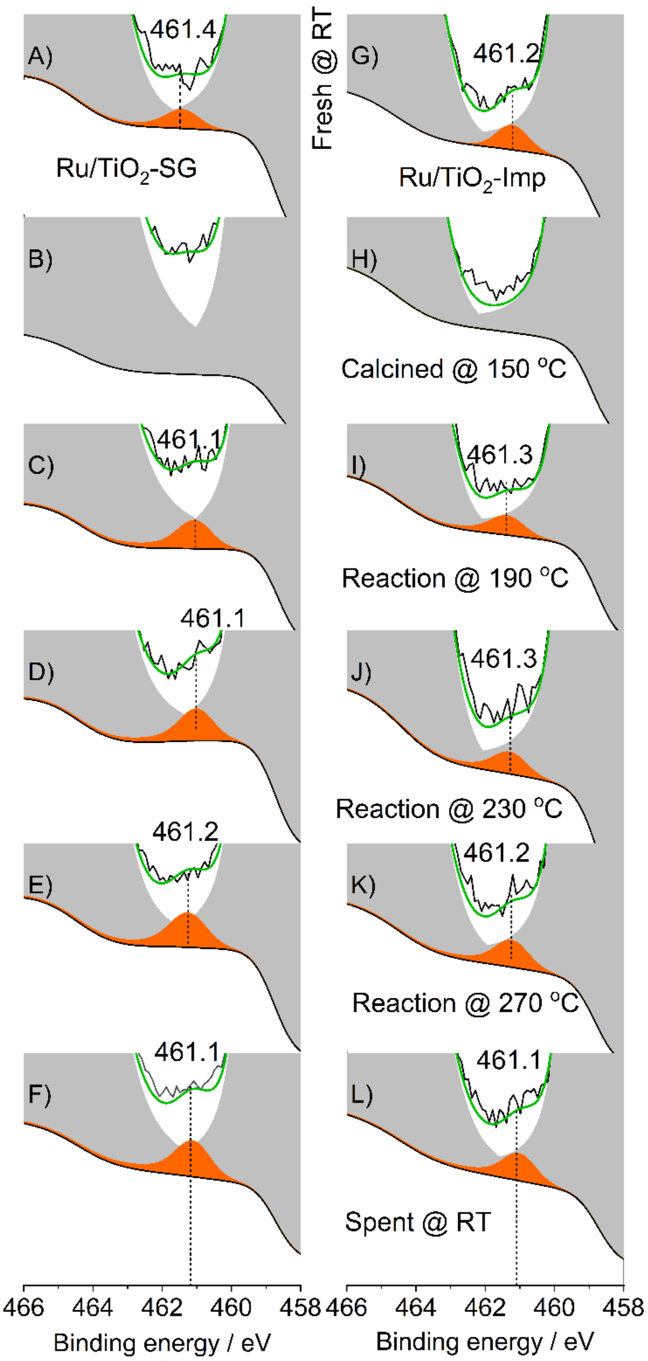

Figure S7. NAP-XPS spectra of Ru/TiO_2-SG and Ru/TiO_2-Imp in the Ti 2p region showing the Ru $3p_{5/2}$ peak recorded at different conditions. Green lines denote the fit sum. Related to STAR Methods. See experimental details in Method Details section. This Figure is also complementary to Figure 7 and Figure S6.

(A, G) Fresh catalysts at RT in He.

(B, H) Oxidation pretreatment before reaction at 150 ℃. 5 vol.% O_2/He.

(C-K) Catalyst at different temperatures. H_2 :CO_2 = 4:1.

(F, L) Spent catalysts at RT in He.

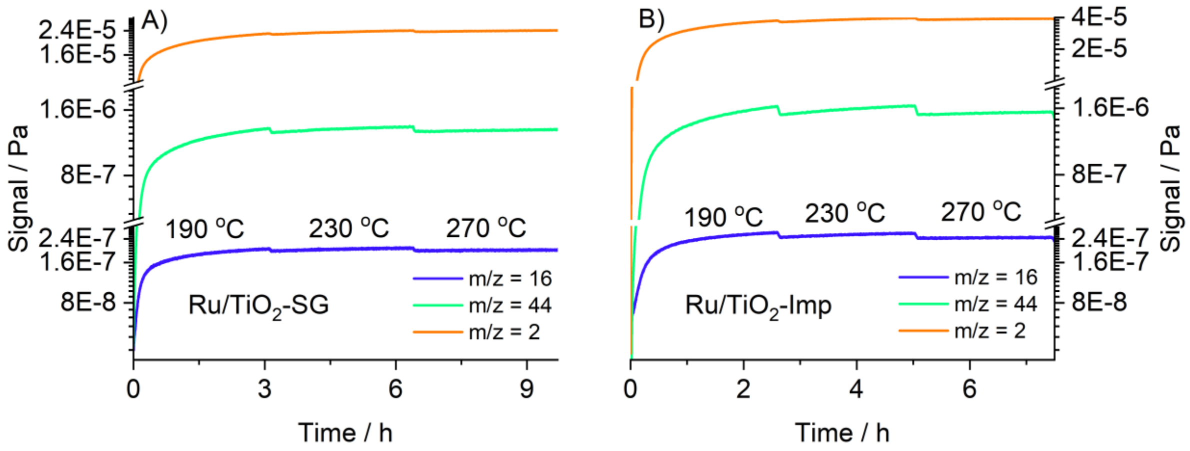

Figure S8. Mass spectra recorded during operando NAP-XPS. Related to STAR Methods. See experimental details in Method Details.

(A) Mass spectra of reaction related species (m/z = 2, H_2; m/z = 16, CH_4; m/z = 44, CO_2) at the outlet of the XP spectrometer at different reaction conditions on Ru/TiO_2-SG: 190 °C to 270 °C, H_2:CO_2 = 4:1.

(B) Mass spectra of reaction related species (m/z = 2, H_2; m/z = 16, CH_4; m/z = 44, CO_2) at the outlet of the XP spectrometer at different reaction conditions on Ru/TiO_2-Imp: 190 °C to 270 °C, H_2:CO_2 = 4:1.

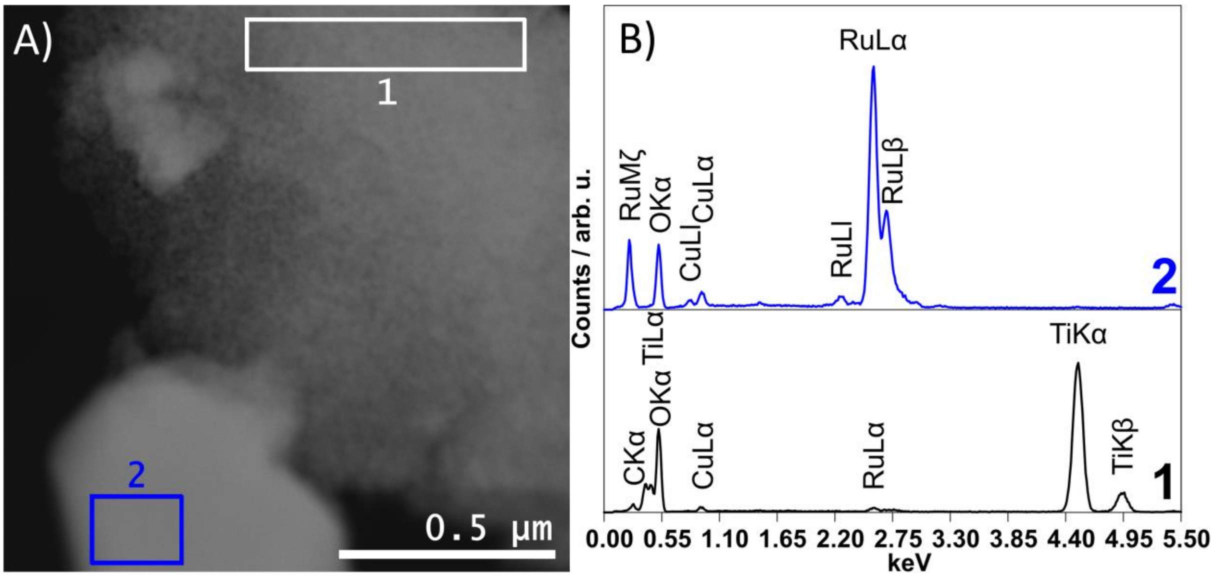

Figure S9. Selected-HAADF and corresponding EDX spectra of fresh Ru/TiO_2-SG. Related to STAR Methods and complementary to Figure 9 A.

A) STEM-HAADF image of fresh Ru/TiO_2-SG.

B) Selected EDX spectra of the marked areas 1 and 2. Area 1 comprises almost exclusively TiO_2 with only little ruthenium, while there is only ruthenium present in area 2. This figure evidence non-uniformity of Ru distribution in the fresh material. See preparation details in Method Details section.

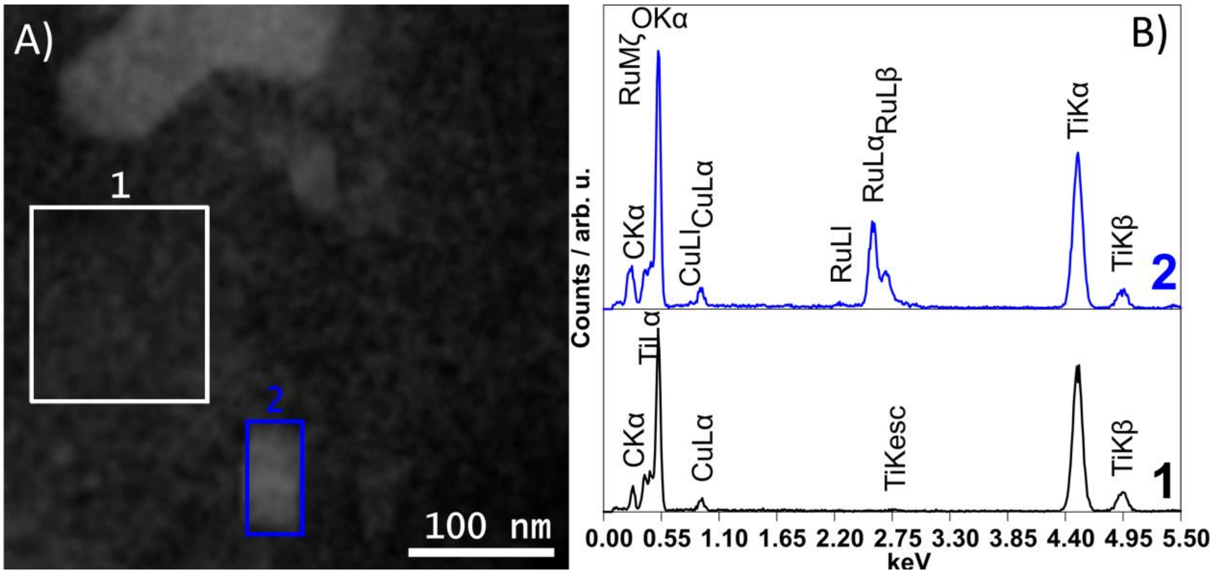

Figure S10. Selected-HAADF and corresponding EDX spectra of fresh Ru/TiO_2-Imp. Related to STAR Methods and complementary to Figure 9 D.

A) STEM-HAADF image of fresh Ru/TiO_2-Imp.

B) Selected EDX spectra of the marked areas 1 and 2. Area 1 comprises exclusively TiO_2 with no ruthenium at all, while there are both, ruthenium and titanium, present in area 2. This figure evidence non-uniformity of Ru distribution in the fresh material. See preparation details in Method Details section.

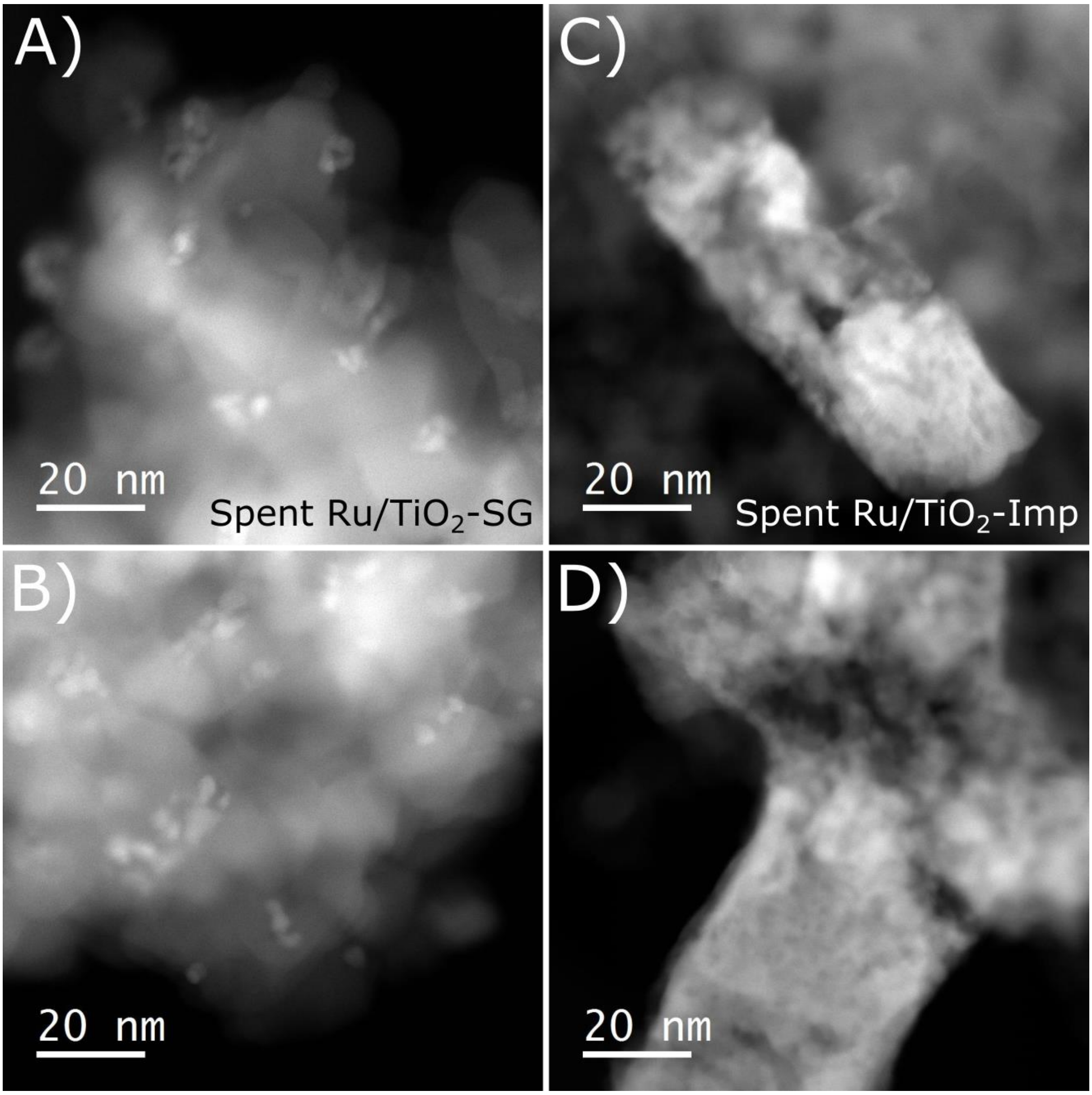

Figure S11. Selected HAADF-STEM micrographs of the spent catalysts. Related to STAR Methods and complementary to Figure 9.
(A, B) Ru/TiO_2-SG.
(C, D) Ru/TiO_2-Imp.
The micrographs were recorded after subjecting the fresh catalysts to the reaction conditions with up to 270 ℃ in H_2:CO_2 = 4:1 (28.8 ml min^{-1} H_2 + 7.2 ml min^{-1} CO_2 + 4 ml min^{-1} Ar).
In spent Ru/TiO_2-SG, the small Ru containing particles are still present, however, changed slightly their appearance and look less spherical and more like being composed of smaller subunits. See experimental details in Method Details section.

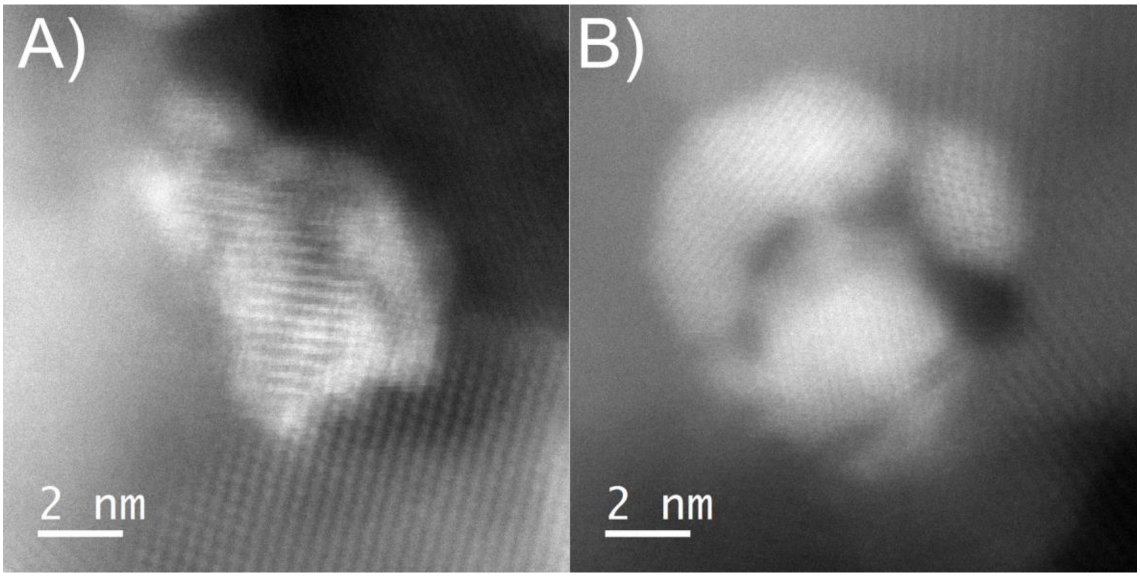

Figure S12. Selected HAADF-STEM micrographs of Ru/TiO$_2$-SG. Related to STAR Methods and complementary to Figure 9 A. See experimental details in Method Details section.
Selected HAADF-STEM images of fresh A) and spent B) Ru/TiO$_2$-SG showing the smaller Ru particles (brighter parts of the images) on TiO$_2$.

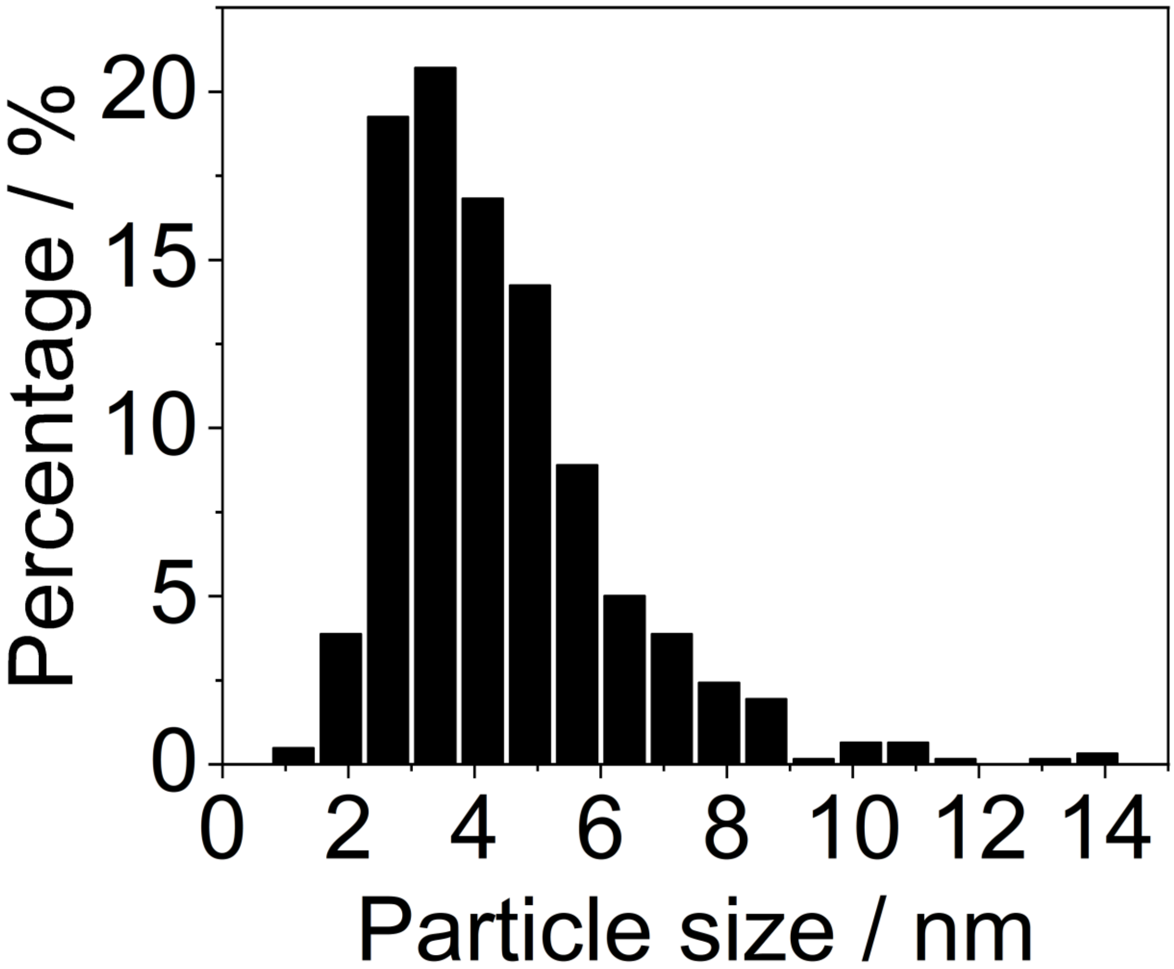

Figure S13. Estimated Ru particle size distribution in the spent Ru/TiO_2-SG. Related to Figure 3 B. Ru particle size distribution estimated from HAADF-STEM images of the spent Ru/TiO_2-SG catalyst. Reaction conditions: Temperature of up to 270 ℃, H_2:CO_2 = 4:1 (28.8 ml min^{-1} H_2 + 7.2 ml min^{-1} CO_2 + 4 ml min^{-1} Ar). The sizes are estimated by evaluation of ca. 300 particles assuming approximately spherical geometry. This Figure is also complementary to Figure 9 E and its information is used in eq. 4, 6 and 7 for calculating the TOF discussed in the Catalytic tests and kinetics analysis section and shown in Figure 3B.

Table S1. Reported activity of CO_2 methanation on different Ru/TiO_2 catalysts synthesized by different methods. This table is complementary to the Catalytic tests shown in Figure 3.

Catalyst	Synthesis method	Reaction temperature	H_2:CO_2	Activity: TOF_{CO2} / Ru mass normalized	Reference[b]
0.9 wt.% Ru/TiO_2 (anatase)	Inorganic sol-gel	140 °C	4:1	1.7 x 10^{-2} s^{-1}	This work
0.9 wt.% Ru/TiO_2 (anatase)	Inorganic sol-gel	160 °C	4:1	3.2 x 10^{-2} s^{-1}	This work
0.9 wt.% Ru/TiO_2 (anatase)	Inorganic sol-gel	170 °C	4:1	9.9 x 10^{-2} s^{-1}	This work
0.9 wt.% Ru/TiO_2 (anatase)	Inorganic sol-gel	190 °C	4:1	0.14 s^{-1}	This work
0.9 wt.% Ru/TiO_2 (anatase)	Inorganic sol-gel	200 °C	4:1	0.46 s^{-1}	This work
0.9 wt.% Ru/TiO_2 (anatase)	Inorganic sol-gel	210 °C	4:1	0.69 s^{-1}	This work
2.43 wt.% Ru (RuO_2/TiO_2-P25)	Colloidal suspension deposition (Annealed at 450 ℃ in static air before reaction)	200 °C	4:1	1.06 x 10^{-4} molCH_4 g_{Ru}^{-1} s^{-1} [a]	Kim et al. (2016)
2.35-2.60 wt.% RuO_2/TiO_2 (50% anatase – 50 % rutile)	Colloidal suspension deposition (Annealed at 450 ℃ in static air before reaction)	Up to 200 °C	4:1	0.057 s^{-1} [a]	Kim et al. (2018)
2.0 wt.% Ru/TiO_2 (P-25) TiO_2	Wetness impregnation	190 °C	5:1	1.0 x 10^{-2} s^{-1}	Abdel-Mageed et al. (2020)
2.1 wt.% Ru/TiO_2(P-90)	Wetness impregnation	190 °C	4:1	6.0 x 10^{-4} molCH_4 g_{Ru}^{-1} s^{-1} [a]	Abdel-Mageed et al. (2021)
2.2 wt.% Ru/TiO_2(P-25)	Wetness impregnation	190 °C	4:1	1.2 x 10^{-3} molCH_4 g_{Ru}^{-1} s^{-1} [a]	Abdel-Mageed et al. (2021)
2.2 wt.% Ru/TiO_2 (P-25)	Colloidal suspension deposition	200 °C	4:1	1.16 x 10^{-4} molCH_4 g_{Ru}^{-1} s^{-1} [a]	Sassoye et al. (2011)
0.8 wt.% Ru/TiO_2 (anatase)	Barrel sputtering	160 °C	4:1	8.5 x 10^{-3} s^{-1}	Abe et al. (2009)
0.5 wt.% Ru/0.2 wt.% Na-TiO_2 (anatase + rutile)	Wet impregnation	250 °C	4:1	0.16 s^{-1}	Panagiotopoulou (2018)
5 wt.% Ru/TiO_2 (rutile)	Wet impregnation	160 °C	4:1	6.0 x 10^{-3} s^{-1}	Lin et al. (2014)
2.39 wt.% Ru/TiO_2	Aqueous colloidal	300 °C	4:1	7.24 x 10^{-2} s^{-1}	Chai et al. (2019)

(anatase 001)					
1.5 wt.% Ru/$TiO_{1.85}N_{0.15}$	Impregnation-reduction	190 °C (under visible light irradiation)	4:1	3.3×10^{-3} s^{-1} [a]	Lin et al. (2017)

[a] Estimated from the respective data.

[b] References in this table are the same as in the main manuscript.
